# Genetic Landscape of Robin Sequence: A Systematic Review

**DOI:** 10.1111/cge.70088

**Published:** 2025-10-12

**Authors:** Shirley van de Velde, Aebele B. Mink van der Molen, Augusta M. A. Lachmeijer, Daan de Leijer, Jeroen J. Smits, Maarten P. G. Massink, Sarah L. Versnel, Marie‐José H. van den Boogaard, Emma C. Paes

**Affiliations:** ^1^ Department of Pediatric Plastic and Reconstructive Surgery University Medical Center Utrecht, Wilhelmina Children's Hospital Utrecht the Netherlands; ^2^ Department of Genetics University Medical Center Utrecht Utrecht the Netherlands; ^3^ University Medical Center Utrecht Utrecht University Utrecht the Netherlands; ^4^ Department of Plastic and Reconstructive Surgery Erasmus University Medical Centre, Sophia Children's Hospital Rotterdam the Netherlands

**Keywords:** genetic testing, genotype–phenotype correlation, Robin sequence, systematic review

## Abstract

Robin sequence (RS) is a congenital condition characterized by micrognathia, glossoptosis, and upper airway obstruction, often occurring with cleft palate and syndromic conditions. The genetic basis of RS is heterogeneous, including monogenic variants and chromosomal rearrangements. This systematic review synthesizes the current genetic landscape of RS, analyzing data from 107 studies that employed various genetic testing methods, including chromosomal microarray (CMA), targeted sequencing, and whole exome sequencing (WES). A distinction is made between genetic variants identified in isolated versus non‐isolated RS. Pathogenic variants in genes as *SOX9*, *SNRPB*, *SATB2*, *TGDS*, *RBM10*, *COL11A1*, and *COL2A1* are frequently identified, many of which are linked to non‐isolated RS. The most common chromosomal aberrations are deletions of 22q11.2 and 18q. Up‐to‐date genetic testing is essential to enable accurate diagnosis and personalized clinical care. With the growing use of whole genome sequencing (WGS) in clinical practice, the need for phenotype‐driven interpretation tools is increasing. Some platforms can prioritize gene relevance based on Human Phenotype Ontology (HPO) terms. Documenting both known and novel RS‐associated genes is therefore crucial to fully realize the diagnostic potential of WGS and support evidence‐based clinical decision‐making.

## Graphical abstract

This systematic review summarizes the genetic landscape of Robin sequence (RS), highlighting key differences between isolated and non‐isolated forms and emphasizing the role of up‐to‐date genetic testing for diagnosis and clinical management.

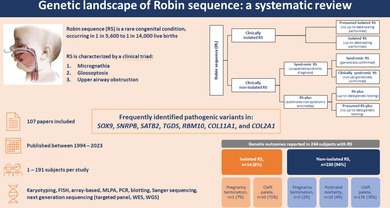

## 1 Introduction

Robin sequence (RS) is a congenital condition characterized by a clinical triad of micrognathia, glossoptosis, and neonatal upper airway obstruction (UAO) [1, 2]. Initially referred to as Pierre Robin Syndrome, it is now known that restricted mandibular growth leads to glossoptosis, resulting in UAO, and in many cases an additional (U‐shaped) cleft palate [3, 4]. Consequently, the condition is currently termed as Robin sequence [5, 6].

**FIGURE 1:**
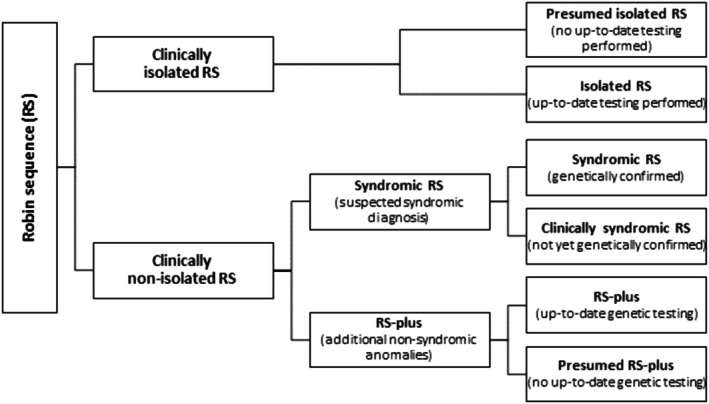
Categorization of RS: (presumed) isolated RS, (clinically) syndromic RS, and (presumed) RS‐plus [8]. [Colour figure can be viewed at wileyonlinelibrary.com]

RS is classified into two main categories: non‐isolated RS and isolated RS, which is based on its genetic and phenotypic manifestations [5, 7]. Non‐isolated RS can be further divided into syndromic RS and RS‐plus (Figure 1). Syndromes most frequently associated with RS include 22q11.2 deletion syndrome, Stickler syndrome, and Treacher Collins syndrome [9, 10, 11]. RS‐plus refers to cases with additional anomalies not associated with recognized syndromes. Isolated RS is defined as RS without a syndromic diagnosis, additional anomalies or developmental disorders (Figure 1).

RS occurs in approximately 1 in 5600 to 1 in 14 000 live births, with studies reporting that 40%–50% of cases are isolated, while 50%–60% are associated with non‐isolated RS [10, 12, 13, 14, 15, 16]. Morbidity and mortality rates in non‐isolated RS are generally higher than in isolated RS due to associated anomalies, which can complicate airway management, feeding, and overall development [17]. Costa et al. and Logjes et al. reported mortality rates of 15%–17% in cases with non‐isolated RS [18, 19]. Infants with non‐isolated RS often require comprehensive multidisciplinary care throughout their lives, and many face ongoing challenges with feeding and respiratory difficulties. For example, they have a fourfold increased risk of postoperative respiratory difficulties following primary cleft palate repair [20]. Therefore, it is important to distinguish between non‐isolated and isolated RS.

While numerous studies have reported on genetic variants associated with RS, a comprehensive overview of literature with genotype–phenotype correlation is lacking. The genetic landscape of RS is complex, particularly in syndromic cases. A genetic diagnosis can have profound implications for clinical management, long‐term prognosis, and recurrence risk in families. Due to recent advancements in genetic testing and the identification of novel genetic variants associated with RS, it is essential to update and integrate this knowledge to pursue best practices in diagnostics and care. This systematic review aims to provide an overview of the genetic variants associated with RS to date, with a focus on their clinical outcomes, as this is crucial for (prenatal) counseling and personalized treatment of infants with RS.

## 2 Methods

This systematic review was performed in accordance with the Preferred Reporting Items for Systematic Reviews and Meta‐Analyses (PRISMA) guidelines.

### 2.1 Search Strategy

A systematic search was conducted using PubMed and Embase (October 10th, 2024), limited to human studies published in Dutch or English. Search terms are listed in the Supporting Information: Content 1. Searches were combined and duplicates were removed. Reference lists of these publications were scanned for additional studies.

### 2.2 Eligibility Criteria and Study Selection

Two reviewers (D.L. and S.V.) independently screened titles, abstracts and full‐texts to identify studies. Studies consisting of a mixed group of subjects, in which those diagnosed with RS could not be separated were excluded. Studies that met one of the exclusion criteria were likewise excluded. The full list of inclusion and exclusion criteria can be found in the Supporting Information: Content 1. Differences between reviewers were reconciled by discussion. If no agreement was reached, the decision to in‐ or exclude was made by a third and fourth reviewer (J.J.S. and E.C.P.).

### 2.3 Quality Assessment

Critical appraisal of the quality of the selected studies was performed using the Critical Appraisal Tools (CAT) of the Joanna Briggs Institute (J.B.I.) for the relevant study design. For the quality assessment, it was necessary to fit the studies into categories; single‐patient descriptions were classified as case reports, multi‐patient descriptions without a control group as case series or cohorts (depending on sample size and degree of systematic data collection), and studies including a comparison group as case–control studies. The studies were assessed independently by two reviewers (D.L. and S.V.). In cases of uncertainty regarding study quality or its impact on inclusion, a final decision was made by a third reviewer (E.C.P.).

### 2.4 Data Extraction and Analysis

Data extracted included study and patient characteristics, RS type, genetic testing methods and outcomes, and inheritance patterns. Relevance to cleft features, mandibular growth, and respiratory outcomes was noted when available. A distinction was made between (presumed) isolated and non‐isolated RS. (Presumed) isolated RS was defined as the presence of the clinical triad (i.e., micrognathia, glossoptosis, and UAO) in the absence of other anomalies, a syndromic diagnosis, intellectual disability, and/or overall developmental delay. Data extraction was independently performed with standardized forms and cross‐checked by DL and SV. A list of RS‐associated genes was compiled and local gene curation criteria were applied (comparison with genetic databases such as *Online Mendelian Inheritance in Man* and *Genomics England PanelApp*) [21, 22].

## 3 Results

### 3.1 Study Selection

The initial search identified 3543 records. After removing duplicates, 2288 studies were screened on title and abstract. Of these, 296 full‐text studies were assessed for eligibility, of which 106 met the criteria. Reference list screening identified one additional study not found in the initial database search. In total, 107 studies were included for critical analysis (Figure 1, Supporting Information: Content 1).

### 3.2 Study Characteristics

The majority of the studies were case reports or case series, with some cohort studies and case–control studies, with a study population ranging from 1 to 191 subjects with Robin sequence (RS). The included studies applied various genome reference builds for variant annotation (hg16, hg18, hg19, and hg38), reflecting the technological developments over time. Regarding the timing of genetic testing, 6 studies reported antenatal analysis, 84 studies reported postnatal or post‐mortem analysis, 14 studies reported both antenatal and postnatal analyses, and 3 studies did not specify the timing. Characteristics of the included studies are presented in Table 1.

**TABLE 1:** Characteristics of studies reporting on genetic variants associated with Robin sequence (RS).

Characteristic	*n* (%)
Included studies in review	107
Study population, total	1010
Subjects with RS within total population	777
Subjects with RS with genetic testing/outcomes	244
Timing of genetic testing	
Antenatal	6 (6)
Postnatal	84 (78)
Antenatal and postnatal	14 (13)
Not reported	3 (3)
Genetic test conducted	
*Antenatal*	
Karyotyping (G‐banding, R‐banding, and chromosome analysis)	13 (12)
FISH	7 (7)
Array‐based (aCGH, CMA, and microarray)	8 (7)
MLPA	2 (2)
*Postnatal*	
Karyotyping (G‐banding, R‐banding, and chromosome analysis)	31 (29)
FISH	26 (24)
Array‐based (aCGH, CMA, microarray, SNP array, and X‐chromosome inactivation array)	45 (42)
MLPA	6 (6)
PCR	8 (7)
Sanger sequencing	39 (36)
Next generation sequencing (targeted panel, WES, and WGS)	41 (38)
Blotting (Western blot and Southern blot)	2 (2)
*Timing not reported*	
Karyotyping (G‐banding and R‐banding)	2 (2)
FISH	1 (1)
Array‐based (aCGH and CMA)	2 (2)
PCR	1 (1)
Blotting (Southern blot)	1 (1)
Human reference genome	
hg16	1 (1)
hg17	0 (0)
hg18	12 (11)
hg19	42 (39)
hg38	1 (1)
Not reported	51 (48)

### 3.3 Quality Assessment

All studies were subjected to a quality assessment (Tables S1–S4, Supporting Information: Content 2). Seventy four (of 107) studies have met 80%–100% of the JBI‐criteria and were classified as low risk of bias, 27 studies met 60%–79% of the JBI‐criteria and were classified as moderate risk of bias, and 6 studies met less than 60% of the JBI‐criteria and were therefore classified as high risk of bias. To summarize, the most frequent forms of bias were not identifying confounders, not reporting on loss to follow‐up, and not describing the matching of groups. No studies were excluded based on quality assessment.

### 3.4 Yield of Genetic Testing

A wide range of genetic testing methodologies was employed across the studies. Several studies utilized multiple genetic testing approaches within the same cohort. One study did not specify the method used [23]. The most frequently reported genetic testing techniques were array‐based (*n* = 55), Sanger sequencing (*n* = 39), FISH (*n* = 34), and WES (*n* = 25).

A comprehensive overview of the genetic variants associated with isolated and non‐isolated RS can be found in Tables 2 and 3, respectively. A detailed overview of the genetic variants found and corresponding phenotype per individual and per study is provided in Supporting Information: Content 3 (Table S5).

**TABLE 2:** Overview of monogenic and chromosomal variants associated with clinically isolated RS.

	Specifications^a^	Phenotype MIM#	Gene MIM#	Condition	References
Monogenic					
*BMPR1B*	NS (SNV)	—	#603248	Isolated RS	[24]
*SLC39A11*	NS (CNV)	—	#616508	Isolated RS	[25]
*SOX9*	NS (SNV/CNV)	—	#608160	Isolated RS	[7, 26, 27, 28, 29]
*TBX22*	NS (SNV)	—	#300307	Isolated RS	[30]
*ZNF804B*	NS (CNV)	—	—	Isolated RS	[31]
Chromosomal					
Deletion	Deletion 2p16; Xp11	—	—	Isolated RS	[32, 33]
Duplication	14q; 22q11	—	—	Isolated RS	[32, 34]
Balanced translocation	t(2;17)	—	—	Isolated RS	[35]

^a^ CNV = copy number variant; NS = not specified; SNV= single nucleotide variant; VUS = variant of uncertain significance.

**TABLE 3:** Overview of monogenic and chromosomal variants associated with clinically non‐isolated RS.

	Specifications^a^	Phenotype MIM#	Gene MIM#	Condition	References
Monogenic					
*ATN1*	NS (repeat region)	#618494	#607642	CHEDDA syndrome	[36]
*ATP2B1*	NS (SNV)	—	#108731	RS‐plus	[37]
*B3GALT6*	NS (SNV)	#271640	#615291	SEMD‐JL	[38]
*BMP2*	NS (CNV)	—	#112261	RS‐plus	[39]
*BMP4*	NS (CNV)	—	#112262	RS‐plus	[40]
*BMPR1B*	NS (SNV/CNV)	—	#603248	RS‐plus	[24]
*COL2A1*	P (SNV/CNV)	#108300	#120140	Stickler syndrome type 1	[14, 41, 42, 43, 44]
*COL2A1*	NS (SNV)	#183900	#120140	Spondylo‐epiphyseal dysplasia congenita	[45]
*COL11A1*	P (SNV/CNV)	#604841	#120280	Stickler syndrome type 2	[14, 33, 46, 47]
*COL11A1*	VUS (SNV)	#604841	#120280	Stickler syndrome type 2	[48]
*CREBBP*	PP	—	#600140	RS‐plus	[49]
*DAAM2*	VUS (CNV)	—	#606627	Non‐isolated RS	[14]
*DPF2*	NS (SNV)	#135900	#601671	Coffin‐Siris syndrome	[50]
*EFTUD2*	NS (NR)	#610536	#603892	Mandibulofacial dysostosis Guion‐Almeida type	[33]
*EIF4A3*	NS (bi‐allelic repeat expansion in 5′ UTR)	#268305	#608546	Richieri‐Costa‐Pereira syndrome	[51]
*FOXC2*	NS (SNV)	#153400	#602402	Lymphedema‐distichiasis syndrome	[52]
*GRHL3*	NS (SNV)	#606713	#608317	Van der Woude syndrome 2	[53]
*GRM4*	NS (CNV)	—	#604100	RS‐plus	[24]
*HIST1H1E*	NS (SNV)	#617537	#142220	Rahman syndrome	[54]
*IRF6*	NS (SNV)	—	#607199	RS‐plus	[55]
*IRF6*	NS (SNV)	#119300	#607199	Van der Woude syndrome	[56]
*KIF15*	NS (SNV)	#619981	#617569	Braddock‐Carey syndrome	[57]
*LMNA*	NS (SNV)	#176670	#150330	Hutchinson‐Gilford progeria syndrome	[58]
*LOXL3*	P (SNV)	—	#607163	RS‐plus	[59]
*MED12*	NS (SNV)	—	#300188	RS‐plus	[60]
*MED13L*	NS (SNV/CNV)	—	#608771	RS‐plus	[61, 62]
*MLL2*	NS (NR)	#147920	#602113	Kabuki syndrome	[33]
*MYMK*	P (SNV)	#254940	#615345	Carey‐Finemann‐Ziter syndrome	[63]
*PGM1*	VUS (CNV)	#614921	#171900	Congenital disorder of glycosylation type It	[33]
*PGM1*	NS (SNV)	#614921	#171900	PGM1 deficiency	[64]
*RBM10*	LP (SNV)	#311900	#300080	TARP syndrome without talipes equinovarus	[65]
*RBM10*	LP/P (SNV/CNV)	#311900	#300080	TARP syndrome	[66, 67, 68, 69, 70, 71, 72]
*RBM10*	NS (SNV)	#311900	#300080	TARP syndrome	[73]
*SATB2*	P (SNV/CNV)	#612313	#608148	Glass syndrome	[14, 23, 33, 74]
*SATB2*	NS	#217980	#608148	Toriello‐Carey syndrome	[75]
*SATB2*	NS	—	#608148	RS‐plus	[76]
*SIX5*	VUS (CNV)	—	#600963	RS‐plus	[59]
*SLC26A2*	NS (SNV)	—	#606718	Signs of DTD and rMED	[77]
*SNRPB*	NS (SNV)	#117650	#182282	Cerebro‐costo‐mandibular syndrome	[78, 79, 80]
*SOX9*	NS (SNV/CNV)	#114290	#608160	Acampomelic campomelic dysplasia	[81, 82, 83, 84, 85, 86]
*SOX9*	VUS	#114290	#608160	Acampomelic campomelic dysplasia	[87]
*SOX9*	NS (CNV)	#114290	#608160	Campomelic dysplasia	[88, 89, 90]
*SOX9*	NS (SNV/CNV) PP (CNV)	—	#608160	RS‐plus	[7, 25, 27, 29, 31, 91, 92, 93]
*TCOF1*	NS (NR)	#154500	#606847	Treacher Collins syndrome	[33]
*TGDS*	NS (SNV/CNV)	#616145	#616146	Catel‐Manzke syndrome	[94]
*TGDS*	LP/P (SNV/CNV)	#616145	#616146	Catel‐Manzke syndrome	[95, 96, 97, 98]
*ZC4H2*	NS (SNV)	#301041	#300897	Female‐restricted Wieacker‐Wolff syndrome	[99]
Chromosomal					
Deletion	Deletion 1p33; 2q13; 2q32; 2q33; 4q34; 6q13; 6q21; 7p14; 7q21; 8p11; 10q26; 11q21; 12q23; 14q22; 16q11; 18p11; 20q13	—	—	RS‐plus	[33, 59, 100, 101, 102, 103, 104, 105, 106, 107, 108]
Deletion	Deletion 1q42; 1q43; 3q22; 3q25; 3q26; 4q31; 5q21; 7q21; 8q24; 9q24; 9q34; 11q23; 13q21; 13q22; 14p11; 12q15; 16p12; 18q21; 21q22	—	—	Non‐isolated RS^b^	[14, 74]
Deletion	Deletion 1p36	#607872	—	1p36 deletion syndrome	[14, 109]
Deletion	Deletion 1q43	#612337	—	1q43q44 deletion syndrome	[110]
Deletion	Microdeletion 2p16.3	#614332	—	2p16.3 deletion syndrome	[33]
Deletion	Microdeletion 3q22.1	—	—	Microdeletion 3q syndrome	[111]
Deletion	Deletion 4p16	#194190	—	Wolf‐Hirschhorn syndrome	[14]
Deletion	Deletion 4q21; 4q32; 4q33; 4q34	—	—	4q deletion syndrome	[74, 107, 112, 113, 114]
Deletion	Deletion 5p15	#123450	—	Cri‐du‐chat syndrome	[14]
Deletion	Deletion 7q11.23	#194050	—	Williams syndrome	[74]
Deletion	Microdeletion 12q13	—	—	Diamond‐Blackfan anemia, Klippel‐Feil syndrome	[115]
Deletion	Microdeletion 16p11.2	#611913	—	16p11.2 deletion syndrome	[33]
Deletion	Deletion 18q22	#601808	—	18q deletion syndrome	[33]
Deletion	Deletion 21q21	#619980	—	Braddock‐Carey syndrome	[116]
Deletion	Deletion 21q22	#619980	—	Braddock‐Carey syndrome	[117]
Deletion	Deletion 22q11.2	#192430	—	22q11.2 deletion syndrome	[14, 33, 74]
Deletion	Deletion 22q12.2	#101000	#607379	Neurofibromatosis type 2	[118, 119]
Deletion	Deletion X	—	—	Turner syndrome	[14]
Deletion	Deletion Yq11 + Deletion 22q11.2	#192430	—	22q11.2 deletion syndrome	[14]
Duplication	Duplication 1p36; 1q23; 2q13; 4q35; 6p25; 9p12; 10q26; 11q23; 12p13; 20p12‐q11	—	—	RS‐plus	[59, 101, 120, 121, 122]
Duplication	Duplication 1p13; 3p14; 4q32; 5q23; 6q15; 7q31; 9p22; 12p13; 12q24; 13q12; 13q32; 15q15; 16p12; 16p13; 19q13; Xp21; Xp22	—	—	Non‐isolated RS^b^	[14, 74]
Duplication	Duplication 1q21	#612475	—	1q21 duplication syndrome	[14, 123]
Duplication	Duplication 15q13	#608636	—	15q11‐q13 duplication syndrome	[14, 74]
Duplication	Duplication 22pter‐q12.3	#115470	—	Cat‐eye syndrome	[124]
Balanced translocation	t(8;17); t(17;18)	—	—	Non‐isolated RS^b^	[14]
Balanced translocation	t(17;20)	—	—	RS‐plus	[125]
Unbalanced translocation	t(1;15); t(2;5); t(3;4); t(3;5); t(6;9); t(11;12); t(11;15); t(13;21); t(14;16); t(13;18); t(X;18)	—	—	Non‐isolated RS^b^	[14, 74]
Unbalanced translocation	t(4;9)	—	—	RS‐plus	[126]
Trisomy	Mosaic trisomy chr3	—	—	Non‐isolated RS	[127]
Trisomy	Mosaic trisomy chr8	—	—	Non‐isolated RS^b^	[74]
Trisomy	Trisomy 12p	—	—	Non‐isolated RS^b^	[74]
Trisomy	Mosaic trisomy chr18	—	—	Non‐isolated RS^b^	[74]
Trisomy	Trisomy chr21	—	—	Non‐isolated RS^b^	[74]

Abbreviations: DTD = Diastrophic Dysplasia; rMED = Recessive form of Multiple Epiphysial Dysplasia; SEMD‐JL = Spondylo‐Epimetaphyseal Dysplasia with Joint Laxity.

^a^ SNV = Single Nucleotide Variant; CNV = Copy Number Variant; *P* = Pathogenic; PP = Possibly Pathogenic; LP = Likely Pathogenic; VUS = Variant of Uncertain Significance; NS = Not Specified.

^b^ Subjects were classified as having non‐isolated Robin sequence; however, the study did not specify the accompanying (craniofacial) anomalies.

### 3.5 Monogenic Causes

Monogenic causes were reported in both isolated and non‐isolated RS, although with distinct distributions (Tables 2 and 3). In isolated RS, only a limited number of genes were identified, including *SOX9* (*n* = 5), and single cases involving *BMPR1B, TBX22, SLC39A11*, and *ZNF8048* [7, 24, 25, 26, 27, 28, 29, 30, 31]. This suggests that the monogenic contribution to isolated RS is relatively restricted, with *SOX9* being the predominant gene.

A much broader genetic spectrum was observed in non‐isolated RS, reflecting its syndromic nature. *SOX9* was again the most frequently implicated gene (*n* = 32), followed by *SNRPB* (*n* = 18), *SATB2* (*n* = 13), *TGDS* (*n* = 12), *RBM10* (*n* = 9), *COL11A1* (*n* = 7), and *COL2A1* (*n* = 6). These genes are associated with recognizable syndromes such as (acampomelic) campomelic dysplasia (*SOX9*), cerebro‐costo‐mandibular syndrome (*SNRPB*), *SATB2*‐associated syndrome (Glass syndrome, *SATB2*), Catel‐Manzke syndrome (*TGDS*), TARP syndrome (*RBM10*), and Stickler syndrome (*COL2A1*, *COL11A1*). In addition, a wide range of less frequently reported genes (*n* = 1–3 per gene) were identified, many of which are primarily linked to broader syndromic or neurodevelopmental phenotypes. Notable monogenic causes included *EIF4A3*, implicated in Richieri‐Costa‐Pereira syndrome, *GRHL3*, associated with orofacial clefting disorders, and *BMP2*, linked to midline clefting and craniofacial dysmorphisms [39, 51, 53]. Variants in *PGM1* were associated with congenital disorders of glycosylation, affecting craniofacial structures [33, 64]. Additionally, variants in *IRF6* were found in cases of non‐isolated RS with cleft palate [55, 56].

### 3.6 Chromosomal Causes

A wide range of chromosomal deletions, duplications, and (unbalanced) translocations were associated with RS in the literature, an overview is provided in Tables 2 and 3. A detailed overview of the variants found per study is provided in Supporting Information: Content 3. The most frequently observed chromosomal abnormality was 22q11.2 deletion. The 22q11 deletion syndrome includes RS along with congenital heart defects and neurodevelopmental delays [14, 33, 74]. 18q deletions were also identified in multiple subjects and studies, contributing to 18q deletion syndrome, which presents with RS, cleft palate, microcephaly, short stature, and growth delays in the subjects identified in the studies [14, 33].

Several chromosomal variants were identified in RS patients that are not classically associated with RS, but rather with broader neurodevelopmental or syndromic phenotypes. For instance, monosomy chromosome X, which underlies Turner syndrome, was reported in one subject with RS [14]. Yet, this is not considered being associated with RS. Similarly, a 16p11.2 microdeletion, known for its strong association with autism spectrum disorder and cognitive delay, was identified in a subject with RS but has not been directly linked to RS in current databases [33]. A subject with a 1p36‐pter deletion was also reported [14, 109]. While this deletion is often associated with a pointed chin, one of the subjects also carried a 19q13.4 deletion, which is more plausibly linked to micrognathia, retrognathia and cleft palate [14]. Additional variants that appear unrelated to RS include duplications of 1q21.1 and 12p13.31 [14, 59, 74, 123]. These duplications are primarily associated with variable developmental and behavioral phenotypes and lack consistent association with RS‐related craniofacial anomalies.

### 3.7 Clinical Course: Isolated Versus Non‐Isolated RS

Across the 107 included studies, genetic testing outcomes were reported for 244 subjects: 14 (6%) with isolated RS and 230 (94%) with non‐isolated RS. Pregnancy was terminated in 6 subjects, including 5 with non‐isolated RS and 1 with isolated RS. In addition, 10 subjects died either in the neonatal period or later in life, all of whom had non‐isolated RS. Of the 13 live‐born subjects with isolated RS, 8 (62%) experienced postnatal respiratory difficulties, 4 (50%) of whom required surgical interventions such as mandibular distraction osteogenesis or tracheostomy. In the other 5 subjects, nothing was reported on UAO. Among the 225 live‐born subjects with non‐isolated RS, 106 (47%) presented with respiratory difficulties. Thirty six (34%) of these subjects necessitated surgical management. It should be noted that not all studies provided information on the presence or absence of airway obstruction, or on how it was managed. Cleft palate was present in 10 (71%) of the isolated RS group and 174 (76%) of the non‐isolated group, with a small number of cases unreported in both groups.

## 4 Discussion

This systematic review provides a comprehensive overview of genetic variants identified in subjects with Robin sequence (RS), distinguishing between isolated and non‐isolated forms, and reporting on phenotypic characteristics and relevant postnatal outcomes such as airway obstruction, feeding difficulties, and global development. A total of 107 studies met the inclusion criteria, comprising cohort studies, case–control studies, case series, and case reports. The findings underscore the genetic heterogeneity of RS, involving both monogenic variants and chromosomal abnormalities.

### 4.1 Identified Monogenic and Chromosomal Causes

Multiple monogenic and chromosomal causes were consistently identified across included studies, with *SOX9*, *SNRPB*, *SATB2*, *TGDS*, *RBM10*, *COL11A1*, and *COL2A1* among the most frequently reported genes. These genes are primarily associated with non‐isolated forms of RS, such as campomelic dysplasia and Stickler syndrome. However, *SOX9* variants have also been reported in isolated cases of RS, suggesting a broader phenotypic spectrum. Among the chromosomal abnormalities, 22q11.2 deletions and deletions on chromosome 18q were most commonly reported.

Interestingly, several included studies focused on cohorts that were not selected exclusively based on an RS phenotype. For instance, in the study by Loewenthal et al., the subjects were initially evaluated due to fasting hypoglycemia and growth retardation [64]; however, all were found to have abnormal palatine structures, including cleft palate, bifid uvula, or RS. In another study by Mouillé et al., subjects were selected based on the presence of a *SATB2* variant, and retrospective analysis revealed six subjects fulfilling the diagnostic criteria for RS [23]. Similarly, in the study by Sahoo et al., subjects were referred for genetic testing because of physical and/or intellectual disabilities and/or dysmorphic features, while Richards et al. included subjects with suspected Stickler syndrome [39, 47].

Although subjects with RS could be clearly identified within these broader cohorts, it is important to note that the genetic variants reported in such studies may not necessarily be causally related to RS itself, but rather to the underlying syndromic or phenotypically overlapping condition. This underlines the importance of applying structured gene curation criteria to distinguish well‐established genotype–phenotype associations from uncertain or incidental findings. Doing so enhances diagnostic accuracy and ensures that genetic counseling is evidence‐based, particularly in a genetically heterogeneous condition like RS.

### 4.2 Clinical Correlation

Importantly, prenatal detection of RS is increasingly feasible through high‐resolution ultrasound and fetal MRI, which may identify features such as micrognathia or glossoptosis. When RS is suspected prenatally, genetic testing such as chromosomal microarray or targeted gene panels may provide early diagnostic clarification. This information can be critical for counseling parents: while some cases may represent isolated RS with a relatively favorable prognosis, others may reflect syndromic forms associated with significant morbidity.

Early recognition of an underlying genetic diagnosis can guide discussions about the likely postnatal course, including risks of airway obstruction, feeding difficulties, and neurodevelopmental outcomes. It also supports practical decision‐making, such as delivery planning in specialized centers with access to neonatal airway expertise, and preparation for interventions in the immediate postnatal period. Nevertheless, prenatal counseling remains challenging, as phenotypic severity is difficult to predict even when a pathogenic variant is identified. For instance, *SOX9* variants may present with isolated RS or with syndromic manifestations such as campomelic dysplasia, leading to very different prognoses. These uncertainties highlight the importance of combining detailed prenatal imaging, family history, and molecular testing results to provide nuanced, individualized counseling for families.

### 4.3 Integrating Modern Genomic Tools Into RS Diagnostic Protocols

Up‐to‐date genetic testing is essential in subjects with RS to enable accurate diagnosis and personalized clinical care (i.e., airway management). Currently, standard genetic workup includes CNV‐analysis and (trio‐based) WES targeting known RS‐associated genes [8]. In specific clinical scenarios, targeted methods such as Sanger sequencing, FISH, or karyotyping may be added. This consideration is particularly relevant when specific familial variants are known.

With recent advancements in genomics, whole genome sequencing (WGS) is increasingly being adopted in both research programs and in routine diagnostics. WGS offers a comprehensive view of the entire genome, covering both coding and non‐coding regions, which significantly enhances the likelihood of identifying causative variants, including deep intronic, repeat, or structural variants not detected by WES. This underscores the importance of having an up‐to‐date and complete overview of all genes currently associated with RS.

In parallel with the implementation of WGS, the use of innovative interpretation platforms is becoming increasingly important. Tools like Franklin (Genoox, Tel Aviv, Israel), which is used at our center, integrate a broad range of genomic databases and provide automated variant annotation and classification according to ACMG guidelines [128]. This facilitates the interpretation of variants by clearly distinguishing between pathogenic, likely pathogenic, variants of uncertain significance (VUS), and benign findings. Because this process is automated and continuously updated with the latest literature and public databases, it helps ensure that clinical teams remain aligned with current genetic knowledge, unless new findings are still unpublished or not yet curated. More importantly, innovative interpretation platforms, such as Franklin, leverage Human Phenotype Ontology (HPO) terms to prioritize genes relevant to the subject's phenotype, improving genotype–phenotype correlation and accelerating the diagnostic process [128]. Such HPO driven analyses may be a valuable addition to the “classic” gene panel approach. These developments highlight that WGS is not only a research tool but increasingly a practical addition to clinical diagnostics, provided that results are interpreted in the context of careful phenotyping.

### 4.4 Limitations

A key limitation is the variability in RS diagnostic criteria across studies. Many included studies were published before 2016 and did not explicitly adhere to the consensus definition of RS (micrognathia, glossoptosis, and UAO) established by Breugem et al. in 2016 [2]. Consequently, there is a risk of heterogeneity in the study population, potentially leading to an over‐ or underestimation of genetic associations.

Furthermore, firm conclusions on the diagnostic yield of genetic testing cannot be drawn. Not all subjects underwent the same or up‐to‐date testing, and some were analyzed with outdated techniques that may not detect subtle abnormalities. In addition, several studies did not report the total number of subjects or specify how many underwent genetic testing. A substantial proportion of the included literature also consisted of case reports describing only single subjects with a genetic variant. Together, these factors restrict the ability to reliably compare the likelihood of identifying a genetic cause in isolated versus non‐isolated RS.

Finally, publication bias may have influenced the findings, as positive genetic findings are more likely to be reported than negative or inconclusive results. Moreover, the studies included in this review exhibited varying levels of methodological quality, as reflected in the risk of bias assessment.

### 4.5 Future Research

Considering these limitations, our review highlights that RS, even when appearing isolated, can have an underlying genetic etiology. This underscores the importance of reevaluating subjects and repeating genetic testing as part of the diagnostic work‐up for RS. Gaining more insight into the phenotypic characteristics associated with different genotypes could contribute to more accurate and efficient (prenatal) counseling, optimized perinatal management, and personalized treatment strategies. Additionally, the role of non‐coding regulatory elements and epigenetic modifications in RS pathogenesis remains largely unexplored and warrants further investigation. A prospective cohort study reevaluating RS subjects without a confirmed genetic diagnosis despite extensive testing is recommended. As genetic technologies advance, such studies will be crucial in identifying novel RS‐related genes. Incorporating WGS and functional studies could provide further insight into the underlying mechanisms of RS, ultimately improving patient outcomes.

## 5 Conclusions

This systematic review highlights the genetic heterogeneity of RS and underscores the importance of distinguishing between isolated and non‐isolated RS. This distinction is essential for accurate variant interpretation and for anticipating associated syndromic features. Beyond theoretical insights, up‐to‐date genetic testing has clear practical implications: prenatally, it can inform counseling, prognosis, and delivery planning; postnatally, it supports airway management, feeding strategies, and developmental follow‐up. As genomic technologies such as open exome and whole genome sequencing become more widely adopted, additional causative variants are expected to be identified. Future studies should adhere to the consensus definition of RS and focus on refining genotype–phenotype correlations, as phenotype‐driven interpretation tools increasingly influence diagnostic workflows. Regularly updating gene panels based on emerging evidence is crucial for improving diagnostic yield. Ultimately, integrating modern genomic approaches into both prenatal and postnatal care pathways is essential for optimizing counseling, perinatal management, and long‐term outcomes in individuals with RS (such as mandibular growth and airway management).

## Author Contributions

E.C.P., M.‐J.H.B. and S.V. conceptualized and designed the study. The systematic literature search was composed by S.V. D.L. and S.V. performed title and abstract screening, full text assessment, risk of bias assessment, data collection, and analysis. J.J.S. and E.C.P. served as third and fourth reviewer in cases where consensus on inclusion or exclusion could not be reached by D.L. and S.V. The first draft of the manuscript was written by S.V., and all authors provided critical feedback on previous versions. All authors read and approved the final manuscript.

## Ethics Statement

The authors have nothing to report.

## Conflicts of Interest

The authors declare no conflicts of interest.

## Supporting information


**Data S1:** cge70088‐sup‐0001‐Supinfo1.pdf.

**Data S2:** cge70088‐sup‐0002‐Supinfo2.pdf.

**Data S3:** cge70088‐sup‐0003‐Supinfo3.pdf.

## Data Availability

The data that support the findings of this study are available from the corresponding author upon reasonable request.
