# Supplementary material for: Genetic Landscape of Robin Sequence: A Systematic Review
Source: Clin Genet. 2025 Oct 12;109(2):218–32. doi: 10.1111/cge.70088 (PMC12779227; doi:10.1111/cge.70088)
Supplement: Supplementary file 1 — Data S1: cge70088‐sup‐0001‐Supinfo1.pdf. [file CGE-109-218-s002.pdf]

Supplemental Data Content 1 – Search and screening

Topic: Genetic variants associated with Robin sequence, found through pre- and postnatal genetic testing

Search terms:

	**Pierre Robin sequence**	**Genetics**	**Genetic testing**	**Genetic variants**
**MeSH**	• Pierre Robin syndrome	• Genetics<br>• Human genetics<br>• Genetics, medical	• Genetic testing<br>• Genetic techniques<br>• High throughput nucleotide sequencing<br>• Whole genome sequencing<br>• Exome sequencing	• Genetic variation<br>• Diagnosis, preimplantation<br>• Prenatal diagnosis<br>• Mutation<br>• Gene duplication<br>• Gene amplification<br>• Genomic instability<br>• Chromosome aberrations<br>• DNA repeat expansion<br>• Base pair mismatch<br>• Frameshift mutation<br>• Gain of function mutation<br>• Point mutation<br>• Sequence deletion<br>• Sequence inversion<br>• Silent mutations<br>• Genetic heterogeneity<br>• Syndrome<br>• Polymorphism, single nucleotide
**Emtree**	• Pierre Robin syndrome	• Genetics<br>• Human genetics<br>• Medical genetics	• Genetic screening<br>• Preimplantation genetic screening<br>• Whole exome sequencing<br>• Whole genome sequencing<br>• High throughput sequencing	• Genetic variation<br>• Preimplantation genetic diagnosis<br>• Prenatal diagnosis<br>• Mutation<br>• Chromosome aberration<br>• Chromosome mutation<br>• Repeat expansion mutation<br>• Base mispairing<br>• Frameshift mutation<br>• Point mutation

				• Silent mutation<br>• Deletion mutation<br>• Gene amplification<br>• Gene mutation<br>• Gene duplication<br>• Genomic instability<br>• Genomic mutation<br>• Induced mutation<br>• Inserted mutation<br>• Genetic heterogeneity<br>• Syndrome<br>• Single nucleotide polymorphism array
**[tiab]**	• Pierre Robin syndrome<br>• Pierre-Robin syndrome<br>• Pierre Robin sequence<br>• Pierre-Robin sequence<br>• Robin sequence<br>• Robin syndrome<br>• Robin anomaly<br>• Robin anomalad<br>• Pierre Robin malformation<br>• Pierre Robin association<br>• PRS<br>• RS<br>• Micrognathia, glossoptosis, and upper airway obstruction	• Genetics<br>• Human genetics<br>• Medical genetics	• Genetic screening<br>• Genetic technic*<br>• Predictive genetic testing<br>• Next generation sequencing<br>• Whole genome sequencing<br>• Whole exome sequencing<br>• High Throughput Nucleotide Sequencing<br>• Next-Generation Sequencing<br>• Sequencing, Next-Generation<br>• Genetic testing panels	• Chromosomal abnormality<br>• Variant of uncertain significance<br>• Gene of uncertain significance<br>• Pathogenic variant<br>• Benign variant<br>• DNA copy number variants<br>• Duplication<br>• Deletion<br>• Inversion<br>• Insertion<br>• Single nucleotide polymorphism<br>• SNP

Search PubMed:

1. Pierre Robin sequence:

((((((((Pierre Robin syndrome[MeSH Terms]) OR (Pierre Robin syndrome[Title/Abstract])) OR (Pierre-Robin syndrome[Title/Abstract])) OR (Pierre Robin sequence[Title/Abstract])) OR (Pierre-Robin sequence[Title/Abstract])) OR (Robin sequence[Title/Abstract])) OR (Robin syndrome[Title/Abstract])) OR (Robin anomal*[Title/Abstract])) OR (Pierre Robin malformation[Title/Abstract])) OR (Pierre Robin association[Title/Abstract])

2. Genetics:

(((((genetics[MeSH Terms]) OR (human genetics[MeSH Terms])) OR (genetics, medical[MeSH Terms])) OR (genetics[Title/Abstract])) OR (human genetics[Title/Abstract])) OR (medical genetics[Title/Abstract])

3. Genetic testing:

((((((((((((genetic testing[MeSH Terms]) OR (genetic testing[Title/Abstract])) OR (genetic techniques[MeSH Terms])) OR (genetic techniques[Title/Abstract])) OR (genetic technic*[Title/Abstract])) OR (high throughput nucleotide sequencing[MeSH Terms])) OR (high throughput nucleotide sequencing[Title/Abstract])) OR (whole genome sequencing[MeSH Terms])) OR (whole genome sequencing[Title/Abstract])) OR (exome sequencing[MeSH Terms])) OR (whole exome sequencing[Title/Abstract])) OR (genetic screening[Title/Abstract])) OR (predictive genetic testing[Title/Abstract])) OR (next generation sequencing[Title/Abstract])

4. Genetic outcomes:

((((((((((genetic variation[MeSH Terms]) OR (genetic variation[Title/Abstract])) OR (diagnosis, preimplantation[MeSH Terms])) OR (preimplantation diagnosis[Title/Abstract])) OR (prenatal diagnosis[MeSH Terms])) OR (prenatal diagnosis[Title/Abstract])) OR (mutation[MeSH Terms])) OR (mutation[Title/Abstract])) OR (syndrome[MeSH Terms])) OR (syndrom*[Title/Abstract])) OR (gene*[Title/Abstract])) OR (chromosom*[Title/Abstract])

**Search**<br>**10-10-2024**	**Query PubMed**	**Results**
#6	**#1 AND #5**	1,442
#5	**#2 OR #3 OR #4**	8,835,410
#4	**((((((((((genetic variation[MeSH Terms]) OR (genetic variation[Title/Abstract])) OR (diagnosis, preimplantation[MeSH Terms])) OR (preimplantation diagnosis[Title/Abstract])) OR (prenatal diagnosis[MeSH Terms])) OR (prenatal diagnosis[Title/Abstract])) OR (mutation[MeSH Terms])) OR (mutation[Title/Abstract])) OR (syndrome[MeSH Terms])) OR (syndrom*[Title/Abstract])) OR (gene*[Title/Abstract])) OR (chromosom*[Title/Abstract])**	8,194,863
#3	**((((((((((((genetic testing[MeSH Terms]) OR (genetic testing[Title/Abstract])) OR (genetic techniques[MeSH Terms])) OR (genetic techniques[Title/Abstract])) OR (genetic technic*[Title/Abstract])) OR (high throughput nucleotide sequencing[MeSH Terms])) OR (high throughput nucleotide sequencing[Title/Abstract])) OR (whole genome sequencing[MeSH Terms])) OR (whole genome sequencing[Title/Abstract])) OR (exome sequencing[MeSH Terms])) OR (whole exome sequencing[Title/Abstract])) OR (genetic screening[Title/Abstract])) OR (predictive genetic testing[Title/Abstract])) OR (next generation sequencing[Title/Abstract])**	2,084,402
#2	**(((((genetics[MeSH Terms]) OR (human genetics[MeSH Terms])) OR (genetics, medical[MeSH Terms])) OR (genetics[Title/Abstract])) OR (human genetics[Title/Abstract])) OR (medical genetics[Title/Abstract])**	478,087
#1	**((((((((Pierre Robin syndrome[MeSH Terms]) OR (Pierre Robin syndrome[Title/Abstract])) OR (Pierre-Robin syndrome[Title/Abstract])) OR (Pierre Robin sequence[Title/Abstract])) OR (Pierre-Robin sequence[Title/Abstract])) OR (Robin sequence[Title/Abstract])) OR (Robin syndrome[Title/Abstract])) OR (Robin anomal*[Title/Abstract])) OR (Pierre Robin malformation[Title/Abstract])) OR (Pierre Robin association[Title/Abstract])**	2,151

Search Embase:

1. Pierre Robin sequence:

'pierre robin syndrome'/exp OR 'pierre robin syndrome':ti,ab,kw OR 'pierre robin sequence':ti,ab,kw OR 'pierre-robin syndrome':ti,ab,kw OR 'pierre-robin sequence':ti,ab,kw OR 'robin sequence':ti,ab,kw OR 'robin syndrome':ti,ab,kw OR 'robin anomal*':ti,ab,kw OR 'pierre robin malformation':ti,ab,kw OR 'pierre robin association':ti,ab,kw

2. Genetics:

'genetics'/exp OR 'human genetics'/exp OR 'medical genetics'/exp OR genetic*:ti,ab,kw OR 'human genetic*':ti,ab,kw OR 'medical genetic*':ti,ab,kw

3. Genetic testing:

'genetic screening'/exp OR 'preimplantation genetic screening'/exp OR 'whole exome sequencing'/exp OR 'whole genome sequencing'/exp OR 'high throughput sequencing'/exp OR 'genetic screening':ti,ab,kw OR 'genetic procedures':ti,ab,kw OR 'genetic technic*':ti,ab,kw OR 'preimplantation genetic screening':ti,ab,kw OR 'whole genome sequencing':ti,ab,kw OR 'whole exome sequencing':ti,ab,kw OR 'high throughput sequencing':ti,ab,kw OR 'predictive genetic testing':ti,ab,kw OR 'next generation sequencing':ti,ab,kw OR 'genetic test*':ti,ab,kw OR 'genetic techniques':ti,ab,kw

4. Genetic outcomes:

'genetic variation'/exp OR 'genetic variation':ti,ab,kw OR 'preimplantation genetic diagnosis'/exp OR 'preimplantation genetic diagnosis':ti,ab,kw OR 'prenatal diagnosis'/exp OR 'prenatal diagnosis':ti,ab,kw OR 'mutation'/exp OR mutation*:ti,ab,kw OR 'syndrome'/exp OR syndrom*:ti,ab,kw OR gene*:ti,ab,kw OR chromosom*:ti,ab,kw

Search<br>10-10-2024	Query Embase	Results
#6	**#1 AND #5**	2,101
#5	**#2 OR #3 OR #4**	10,882,859
#4	**'genetic variation'/exp OR 'genetic variation':ti,ab,kw OR 'preimplantation genetic diagnosis'/exp OR 'preimplantation genetic diagnosis':ti,ab,kw OR 'prenatal diagnosis'/exp OR 'prenatal diagnosis':ti,ab,kw OR 'mutation'/exp OR mutation*:ti,ab,kw OR 'syndrome'/exp OR syndrom*:ti,ab,kw OR gene*:ti,ab,kw OR chromosom*:ti,ab,kw**	10,406,101
#3	**'genetic screening'/exp OR 'preimplantation genetic screening'/exp OR 'whole exome sequencing'/exp OR 'whole genome sequencing'/exp OR 'high throughput sequencing'/exp OR 'genetic screening':ti,ab,kw OR 'genetic procedures':ti,ab,kw OR 'genetic technic*':ti,ab,kw OR 'preimplantation genetic screening':ti,ab,kw OR 'whole genome sequencing':ti,ab,kw OR 'whole exome sequencing':ti,ab,kw OR 'high throughput sequencing':ti,ab,kw OR 'predictive genetic testing':ti,ab,kw OR 'next generation sequencing':ti,ab,kw OR 'genetic test*':ti,ab,kw OR 'genetic techniques':ti,ab,kw**	476,114
#2	**'genetics'/exp OR 'human genetics'/exp OR 'medical genetics'/exp OR genetic*:ti,ab,kw OR 'human genetic*':ti,ab,kw OR 'medical genetic*':ti,ab,kw**	2,743,865
#1	**'pierre robin syndrome'/exp OR 'pierre robin syndrome':ti,ab,kw OR 'pierre robin sequence':ti,ab,kw OR 'pierre-robin syndrome':ti,ab,kw OR 'pierre-robin sequence':ti,ab,kw OR 'robin sequence':ti,ab,kw OR 'robin syndrome':ti,ab,kw OR 'robin anomal*':ti,ab,kw OR 'pierre robin malformation':ti,ab,kw OR 'pierre robin association':ti,ab,kw**	3,291

Inclusion criteria:

- (Pierre) Robin sequence
- Genetic testing is performed, antenatally or postnatally

Exclusion criteria:

- Other language than Dutch or English
- Abstract, editorials, review, survey, letter to the editor, expert opinion, comments on article
- Animal studies
- Association studies
- No full text available
- Other outcomes than genetic variations

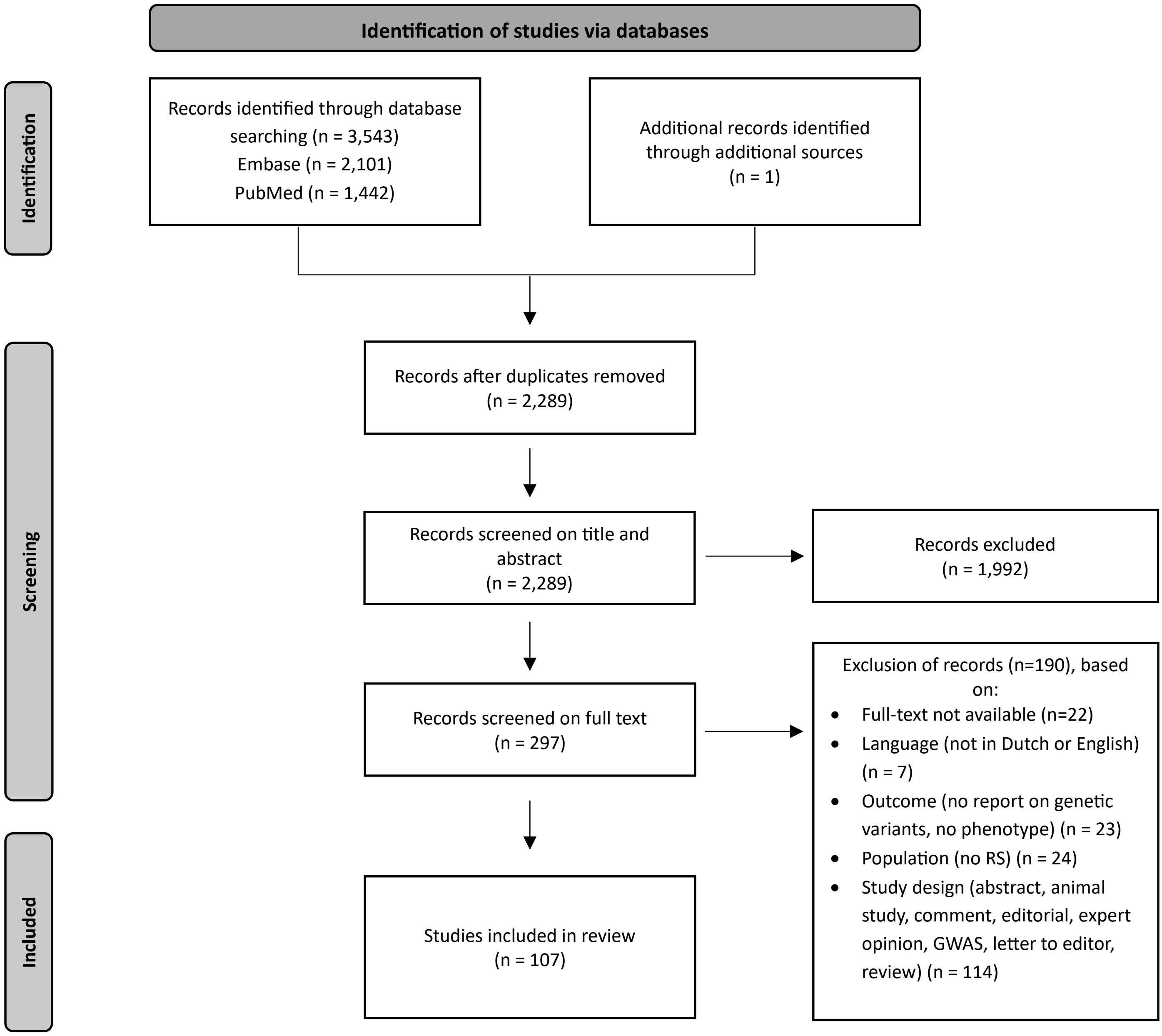

Figure 1. PRISMA flow diagram of the study selection process
