# Supplementary material for: Genetic Landscape of Robin Sequence: A Systematic Review
Source: Clin Genet. 2025 Oct 12;109(2):218–32. doi: 10.1111/cge.70088 (PMC12779227; doi:10.1111/cge.70088)
Supplement: Supplementary file 2 — Data S2: cge70088‐sup‐0002‐Supinfo2.pdf. [file CGE-109-218-s003.pdf]

Supplemental Data Content 2 – Quality assessment

Supplemental Table 1. Quality assessment of papers: case reports

Reference	1. Patient's demographic characteristics described?	2. Patient's history described and presented as a timeline?	3. Current clinical condition of patient on presentation described?	4. Diagnostic tests or assessment methods and results described?	5. Intervention or treatment procedure described?	6. Post-intervention clinical condition described?	7. Adverse (harms) or unanticipated events described?	8. Does case report provide takeaway lessons?	Score	Risk of bias
Aboura et al., 2002 [23]	Yes	Yes	Yes	Yes	Not applicable	Not applicable	Not applicable	Yes	5/5 (100%)	Low
Amarillo et al., 2013 [24]	Yes	Yes	Yes	Yes	Not applicable	Not applicable	Not applicable	Yes	5/5 (100%)	Low
Antwi et al., 2018 [25]	Yes	Yes	Yes	Yes	Not applicable	Not applicable	Not applicable	Yes	5/5 (100%)	Low
Camacho et al., 2020 [33]	Yes	Yes	Yes	Yes	Not applicable	Not applicable	Not applicable	Yes	5/5 (100%)	Low
Capkova et al., 2017 [34]	Yes	Yes	Yes	Yes	Not applicable	Not applicable	Not applicable	Yes	5/5 (100%)	Low
Daicheng et al., 2022 [36]	Yes	Yes	Yes	Yes	Not applicable	Not applicable	Not applicable	Yes	5/5 (100%)	Low

Davidson et al., 2012 [37]	Yes	Yes	Yes	Yes	Not applicable	Not applicable	Not applicable	Yes	5/5 (100%)	Low
Dupont et al., 2013 [40]	Yes	Yes	Yes	Yes	Not applicable	Not applicable	Not applicable	Yes	5/5 (100%)	Low
Fukami et al., 2012 [42]	Yes	Yes	Yes	Yes	Not applicable	Not applicable	Not applicable	Yes	5/5 (100%)	Low
Gerard-Blanluet et al., 2007 [43]	Yes	Yes	Yes	Yes	Not applicable	Not applicable	Not applicable	Yes	5/5 (100%)	Low
Gerth-Kahlert et al., 2011 [44]	Yes	Yes	Yes	Yes	Not applicable	Not applicable	Not applicable	Yes	5/5 (100%)	Low
Gopa-kumar et al., 2014 [46]	Yes	Yes	Yes	Yes	Not applicable	Not applicable	Not applicable	No	4/5 (80%)	Low
Gripp et al., 2011 [49]	Yes	Yes	Yes	Yes	Not applicable	Not applicable	Not applicable	Yes	5/5 (100%)	Low
Houdayer et al., 2001 [50]	Yes	Yes	Yes	Yes	Not applicable	Not applicable	Not applicable	Yes	5/5 (100%)	Low
Higuchi et al., 2017 [51]	Yes	Yes	Yes	Yes	Not applicable	Not applicable	Not applicable	Yes	5/5 (100%)	Low

Højland et al., 2018 [52]	Yes	Yes	Yes	Yes	Not applicable	Not applicable	Not applicable	Yes	5/5 (100%)	Low
Hui et al., 2020 [54]	Yes	Yes	Yes	Yes	Not applicable	Not applicable	Not applicable	Yes	5/5 (100%)	Low
Indugula et al., 2022 [55]	Yes	Yes	Yes	Yes	Not applicable	Not applicable	Not applicable	Yes	5/5 (100%)	Low
Izumi et al., 2012 [56]	Yes	Yes	Yes	Yes	Not applicable	Not applicable	Not applicable	Yes	5/5 (100%)	Low
Izumi et al., 2015 [57]	Yes	Yes	Yes	Yes	Not applicable	Not applicable	Not applicable	Yes	5/5 (100%)	Low
Jiang et al., 2020 [61]	Yes	Yes	Yes	Yes	Not applicable	Not applicable	Not applicable	Yes	5/5 (100%)	Low
Keeling et al., 2001 [63]	Yes	Yes	Yes	Yes	Not applicable	Not applicable	Not applicable	Yes	5/5 (100%)	Low
Kekis et al., 2016 [64]	Yes	Yes	Yes	Yes	Not applicable	Not applicable	Not applicable	Yes	5/5 (100%)	Low
Knapp et al., 2019 [65]	Yes	Yes	Yes	Yes	Not applicable	Not applicable	Not applicable	Yes	5/5 (100%)	Low

Kohmoto et al., 2016 [66]	Yes	Yes	Yes	Yes	Not applicable	Not applicable	Not applicable	Yes	5/5 (100%)	Low
Kramer et al., 2000 [67]	Yes	Yes	Yes	Yes	Not applicable	Not applicable	Not applicable	Yes	5/5 (100%)	Low
Kylat, 2018 [69]	Yes	Yes	Yes	Yes	Not applicable	Not applicable	Not applicable	Yes	5/5 (100%)	Low
Lane et al., 2023 [70]	Yes	Yes	Yes	Yes	Not applicable	Not applicable	Not applicable	Yes	5/5 (100%)	Low
Lecointre et al., 2009 [71]	No	Yes	Yes	Yes	Not applicable	Not applicable	Not applicable	Yes	4/5 (80%)	Low
Manotas et al., 2021 [75]	No	Yes	Yes	Yes	Not applicable	Not applicable	Not applicable	Yes	4/5 (80%)	Low
Martinet et al., 2006 [76]	Yes	Yes	Yes	Yes	Not applicable	Not applicable	Not applicable	Yes	5/5 (100%)	Low
Miller et al., 2020 [78]	No	Yes	Yes	Yes	Not applicable	Not applicable	Not applicable	Yes	4/5 (80%)	Low
Moog et al., 2001 [79]	Yes	Yes	Yes	Yes	Not applicable	Not applicable	Not applicable	Yes	5/5 (100%)	Low

Morozumi et al., 2018 [80]	No	Yes	Yes	Yes	Not applicable	Not applicable	Not applicable	Yes	4/5 (80%)	Low
Murtaza et al., 2021 [82]	No	Yes	No	Yes	Not applicable	Not applicable	Not applicable	Yes	3/5 (60%)	Moderate
Nunes et al., 21994 [84]	Yes	Yes	Yes	Yes	Not applicable	Not applicable	Not applicable	Yes	5/5 (100%)	Low
Omorodion et al., 2023	No	Yes	Yes	Yes	Not applicable	Not applicable	Not applicable	Yes	4/5 (80%)	Low
Õunap et al., 2005 [86]	No	Yes	Yes	Yes	Not applicable	Not applicable	Not applicable	Yes	4/5 (80%)	Low
Owczarek-Lipska et al., 2022 [87]	Yes	Yes	Yes	Yes	Not applicable	Not applicable	Not applicable	Yes	5/5 (100%)	Low
Parmeggiani et al., 2017 [88]	Yes	Yes	Yes	Yes	Not applicable	Not applicable	Not applicable	Yes	5/5 (100%)	Low
Pferdehirt et al., 2015 [90]	Yes	Yes	Yes	Yes	Not applicable	Not applicable	Not applicable	Yes	5/5 (100%)	Low
Preiksaitiene et al., 2016 [91]	No	Yes	Yes	Yes	Not applicable	Not applicable	Not applicable	Yes	4/5 (80%)	Low

Ramieri et al., 2011 [94]	No	Yes	Yes	Yes	Not applicable	Not applicable	Not applicable	Yes	4/5 (80%)	Low
Robbins et al., 2018 [96]	No	Yes	Yes	Yes	Not applicable	Not applicable	Not applicable	Yes	4/5 (80%)	Low
Roberti et al., 2018 [97]	No	Yes	Yes	Yes	Not applicable	Not applicable	Not applicable	Yes	4/5 (80%)	Low
Saito et al., 2022 [100]	Yes	Yes	Yes	Yes	Not applicable	Not applicable	Not applicable	Yes	5/5 (100%)	Low
Salinero et al., 2020 [101]	No	Yes	Yes	Yes	Not applicable	Not applicable	Not applicable	Yes	4/5 (80%)	Low
Sangsin et al., 2016 [103]	Yes	Yes	Yes	Yes	Not applicable	Not applicable	Not applicable	Yes	5/5 (100%)	Low
Schoner et al., 2017 [104]	Yes	Yes	Yes	Yes	Not applicable	Not applicable	Not applicable	Yes	5/5 (100%)	Low
Sismani et al., [105]	Yes	Yes	Yes	Yes	Not applicable	Not applicable	Not applicable	Yes	5/5 (100%)	Low
Sleiman et al., 2017 [106]	Yes	Yes	Yes	Yes	Not applicable	Not applicable	Not applicable	Yes	5/5 (100%)	Low

Smyk et al., 2015 [107]	No	Yes	Yes	Yes	Not applicable	Not applicable	Not applicable	Yes	4/5 (80%)	Low
Sun et al., 2014 [110]	No	Yes	Yes	Yes	Not applicable	Not applicable	Not applicable	Yes	4/5 (80%)	Low
Sun et al., 2022 [111]	Yes	Yes	Yes	Yes	Not applicable	Not applicable	Not applicable	Yes	5/5 (100%)	Low
Take-nouchi et al., 2014 [112]	No	Yes	Yes	Yes	Not applicable	Not applicable	Not applicable	Yes	4/5 (80%)	Low
Tanpai-boon et al., 2010 [114]	Yes	Yes	Yes	No	Not applicable	Not applicable	Not applicable	Yes	4/5 (80%)	Low
Taub et al., 2012 [115]	Yes	Yes	Yes	Yes	Not applicable	Not applicable	Not applicable	Yes	5/5 (100%)	Low
Tegay et al., 2009 [116]	Yes	Yes	Yes	Yes	Not applicable	Not applicable	Not applicable	Yes	5/5 (100%)	Low
Utami et al., 2014 [118]	No	Yes	Yes	Yes	Not applicable	Not applicable	Not applicable	Yes	4/5 (80%)	Low
Walters-Sen et al., 2014 [121]	No	Yes	Yes	Yes	Not applicable	Not applicable	Not applicable	Yes	4/5 (80%)	Low

Xu et al., 2023 [123]	Yes	Yes	Yes	Yes	Not applicable	Not applicable	Not applicable	Yes	5/5 (100%)	Low
Yap et al., 2023 [125]	Yes	Yes	Yes	Yes	Not applicable	Not applicable	Not applicable	Yes	5/5 (100%)	Low
Yekula et al., 2020 [126]	No	Yes	Yes	Yes	Not applicable	Not applicable	Not applicable	Yes	4/5 (80%)	Low

Supplemental Table 2. Quality assessment of papers: case series

Reference	1. Clear inclusion criteria?	2. Condition measured in a standard, reliable way for all participants?	3. Valid methods used for identification of the condition?	4. Consecutive inclusion of participants?	5. Complete inclusion of participants?	6. Clear reporting of the demo-graphics of participants?	7. Clear reporting of clinical information of participants?	8. Outcomes of genetic testing in cases clearly reported?	9. Clear reporting of the presenting site/clinic demo-graphic information?	10. Appropriate statistical analysis?	Score	Risk of bias
Bacrot et al., 2014 [26]	Yes	Yes	Yes	No	Unclear	Yes	Yes	Yes	Yes	Not applicable	7/9 (78%)	Moderate
Bertola et al., 2017 [29]	Yes	Yes	Yes	No	Unclear	Yes	Yes	Yes	Yes	Not applicable	7/9 (78%)	Moderate
Bhoj et al., 2013 [30]	Yes	Yes	Yes	No	Unclear	Yes	Yes	Yes	No	Not applicable	6/9 (67%)	Moderate
Boschann et al., 2020 [31]	Yes	Yes	Yes	No	Unclear	Yes	Yes	Yes	No	Not applicable	6/9 (67%)	Moderate
Braddock et al., 2016 [32]	Yes	Yes	Yes	No	Unclear	No	Yes	Yes	No	Not applicable	5/9 (56%)	High
Castori et al., 2015 [35]	Yes	Yes	Yes	No	Unclear	Yes	Yes	Yes	No	Not applicable	6/9 (67%)	Moderate

De Lonlay-Debeney et al., 1998 [38]	Yes	Yes	Yes	No	Unclear	Yes	Yes	Yes	No	Not applicable	6/9 (67%)	Moderate
Ehmke et al., 2014 [41]	Yes	Yes	Yes	No	Unclear	Yes	Yes	Yes	No	Not applicable	6/9 (67%)	Moderate
Gomez-Ospina and Bernstein, 2016 [45]	Yes	Yes	Yes	Yes	No	Yes	Yes	Yes	Yes	Yes	9/10 (90%)	Low
Gordon et al., 2014 [47]	Yes	Unclear	Unclear	No	Unclear	No	Yes	Yes	No	Not applicable	3/9 (33%)	High
Gordon et al., 2017 [48]	Yes	Yes	Yes	No	Unclear	Yes	Yes	Yes	No	Not applicable	6/9 (67%)	Moderate
Honey, 2016 [53]	Yes	Yes	Yes	No	Unclear	Yes	Yes	Yes	No	Not applicable	6/9 (67%)	Moderate
Jakobsen et al., 2007 [58]	Yes	Yes	Yes	Yes	Unclear	Yes	Yes	Yes	No	Yes	8/10 (80%)	Low
Jamshidi et al., 2004 [59]	Yes	Yes	Yes	No	Unclear	Yes	Yes	Yes	No	Not applicable	6/9 (67%)	Moderate
Jezela-Stanek et al., 2009 [60]	No	Unclear	Unclear	No	Unclear	Yes	Yes	Yes	No	Not applicable	3/9 (33%)	High

Johnston et al., 2013 [62]	Yes	Yes	Yes	No	Unclear	Yes	Yes	Yes	Yes	Not applicable	8/9 (89%)	Low
Kumps et al., 2021 [68]	Yes	Yes	Yes	Yes	No	No	Yes	Yes	No	Not applicable	6/9 (67%)	Moderate
Loewenthal et al., 2015 [72]	Yes	Yes	Yes	Yes	Yes	Yes	Yes	Yes	Yes	Not applicable	9/9 (100%)	Low
Lumaka et al., 2012 [73]	Yes	Yes	Yes	No	Unclear	Yes	Yes	Yes	No	Not applicable	6/9 (67%)	Moderate
Lynch et al., 2014 [74]	Yes	Yes	Yes	No	Unclear	Yes	Yes	Yes	Yes	Not applicable	7/9 (78%)	Moderate
Micale et al., 2020 [77]	Yes	Yes	Yes	No	Unclear	Yes	Yes	Yes	No	Not applicable	6/9 (67%)	Moderate
Nelson et al., 2011 [83]	Yes	Yes	Yes	No	Unclear	Yes	Yes	Yes	No	Not applicable	6/9 (67%)	Moderate
Pengelly et al., 2015 [89]	Yes	Unclear	No	No	Unclear	Yes	Yes	Yes	Yes	Not applicable	5/9 (56%)	High
Prescott et al., 2016 [92]	Yes	Yes	Yes	No	Unclear	Yes	Yes	Yes	No	Not applicable	6/9 (67%)	Moderate

Rainger et al., 2014 [93]	Yes	Yes	Yes	No	Unclear	Yes	Yes	Yes	No	Not applicable	6/9 (67%)	Moderate
Richards et al., 2013 [95]	Yes	Yes	Yes	No	Unclear	No	Yes	Yes	Yes	Not applicable	6/9 (67%)	Moderate
Rossi et al., 2009 [98]	Yes	Yes	Yes	No	Unclear	No	Yes	Yes	Yes	Not applicable	6/9 (67%)	Moderate
Sahoo et al., 2011 [99]	Yes	Unclear	Unclear	No	Unclear	Yes	Yes	Yes	No	Not applicable	4/9 (44%)	Low
Sanchez-Castro et al., 2013 [102]	Yes	Unclear	No	No	Unclear	Yes	Yes	Yes	No	Not applicable	4/9 (44%)	Low
Sood et al., 2021 [108]	Yes	Yes	Unclear	Yes	Yes	Yes	Yes	Yes	Yes	Not applicable	8/9 (89%)	Low
Suemori et al., 2013 [109]	Yes	Yes	Yes	No	Unclear	Yes	Yes	Yes	No	Not applicable	6/9 (67%)	Moderate
Takeshita et al., 2017 [113]	Yes	Unclear	Unclear	No	Unclear	Yes	Yes	Yes	No	Not applicable	4/9 (44%)	Low
Tooley et al., 2016 [117]	Yes	No	Unclear	No	Unclear	Yes	Yes	Yes	No	Not applicable	4/9 (44%)	Low

VanLanding-ham et al., 2008 [119]	Yes	Yes	Yes	No	Unclear	Yes	Yes	Yes	No	Not applicable	6/9 (67%)	Moderate
Velagaleti et al., 2005 [120]	Yes	Yes	Yes	No	Unclear	Yes	Yes	Yes	No	Not applicable	6/9 (67%)	Moderate
Zechi-Ceide et al., 2013 [127]	Yes	Yes	Yes	No	Unclear	Yes	Yes	Yes	No	Not applicable	6/9 (67%)	Moderate

Supplemental Table 3. Quality assessment of papers: cohort studies

Reference	1. Similarity of groups and recruited from same population?	2. Exposures measured similarly to assign people to both exposed and unexposed groups?	3. Exposure measured in a valid and reliable way?	4. Con-founding factors identified?	5. Strategies to deal with con-founding factors?	6. Groups/ Partici-pants free of the outcome at the start?	7. Outcomes measured in a valid and reliable way?	8. Follow up time reported and sufficient?	9. Follow up complete? Reasons to loss to follow up described?	10. Strategies to address incomplete follow up?	11. Appro-priate statistical analysis?	Score	Risk of bias
Basart et al., 2015 [13]	Yes	Not applicable	Not applicable	No	Not applicable	Not applicable	Yes	Yes	No	No	Not applicable	3/6 (50%)	High
Basha et al., 2018 [27]	Yes	Not applicable	Not applicable	No	Not applicable	Not applicable	Yes	Yes	Yes	Not applicable	Not applicable	4/5 (80%)	Low
Demeer et al., 2018 [39]	Yes	Not applicable	Not applicable	No	Not applicable	Not applicable	Yes	Unclear	Yes	Not applicable	Not applicable	3/5 (60%)	Moderate
Mouillé et al., 2022 [81]	Yes	Not applicable	Not applicable	No	Not applicable	Not applicable	Yes	Yes	Yes	Not applicable	Yes	5/6 (83%)	Low
Weaver et al., 2022 [122]	Yes	Not applicable	Not applicable	Yes	Yes	Not applicable	Yes	Yes	No	No	Yes	5/8 (63%)	Moderate
Xu et al., 2016 [7]	Yes	Not applicable	Not applicable	No	Not applicable	Not applicable	Yes	Unclear	Yes	Not applicable	Not applicable	3/5 (60%)	Moderate

Supplemental Table 4. Quality assessment of papers: case-controls

Reference	1. Groups comparable?	2. Cases and controls matched appro-priately?	3. Same criteria for identifi-cation?	4. Exposure measured in valid and reliable way?	5. Exposure measured in same way for cases/ controls?	6. Con-founding factors?	7. Strategies to deal with confounding factors?	8. Outcomes assessed in valid and reliable way?	9. Exposure period of interest long enough?	10. Appropriate statistical analysis?	Score	Risk of bias
Benko et al., 2009 [28]	Unclear	Unclear	Yes	Not applicable	Not applicable	No	Not applicable	Yes	Not applicable	Not applicable	2/5 (40%)	High
Yang et al., 2017 [124]	Yes	Yes	Yes	Not applicable	Not applicable	No	Not applicable	Yes	Not applicable	Not applicable	4/5 (80%)	Low
