# Supplementary material for: Genetic Landscape of Robin Sequence: A Systematic Review
Source: Clin Genet. 2025 Oct 12;109(2):218–32. doi: 10.1111/cge.70088 (PMC12779227; doi:10.1111/cge.70088)
Supplement: Supplementary file 3 — Data S3: cge70088‐sup‐0003‐Supinfo3.pdf. [file CGE-109-218-s001.pdf]

Supplemental Table 5. Detailed overview of monogenic and chromosomal variants associated with RS, per study

Reference	Gene (NM_ number) /chromosomal aberration	Variant	Classification of variant†	Phenotype MIM#	Gene MIM#	Phenotypes			Clinical course	Associated syndrome
						Mandible	Palate	Other‡		
Aboura et al., 2002	Duplication	Partial trisomy 1q, de novo dup 1q23.1q31.1	NS	-	-	Micro-retrognathia	Posterior cleft of soft palate	Present: Small and bifid tongue, brachycephaly, short neck, broad nostrils, short oblique palpebral fissures, low-set ears, bilateral and symmetric camptodactyly (hands), slightly wide-set nipples, decreased number of motor neurons showing apoptotic features in anterior horn	Termination of pregnancy at 31 weeks gestation	
Amarillo et al., 2013	*SOX9*	623-638 kb microdeletion in 17q24.3 –725 kb upstream (5’) of *SOX9*	NS	-	#608160	Micrognathia	U-shaped cleft	Absent, classified as isolated RS	Respiratory distress requiring bilateral MDO, and feeding difficulties. Delayed speech development. Normal overall development.	

Antwi et al., 2018	*SOX9*	Unbalanced translocation, t(6;17) (p21.1;q24.3)	NS	#114290	#608160	Mild retrognathia	Cleft	Present: Hypoplastic maxilla, hypertelorism, leftward deviation soft nasal septum, 45° vertical angulation of the skull base, hypoplastic C6 vertebral body, exaggerated cervical lordosis and thoracic kyphosis with severe canal stenosis (T1-4), hypoplastic right ovary, angulated left femoral shaft, hypoplastic pubic bones and absent ossification of the right talus, and diffuse ventriculo-megaly	Respiratory distress at birth requiring intubation and NICU admission. Readmission at 2 months of age: mechanical ventilation and later tracheostomy for ventilator support	Campomelic dysplasia

Bacrot et al., 2014	*SNRPB* (NM_198216, NM_003091)	g.2447951C>G c.166G>C p.Gly56Arg	NS	#117650	#182282	Micro-retrognathia	NR	Present: Posterior rib gap defects	Neonatal hospitalization, multiple orofacial interventions. Conductive hearing loss	Cerebro-costo-mandibular syndrome
	SNRPB (NM_198216, NM_003091)	g.2447952C>G c.165G>C p.Arg55Ser	NS	#117650	#182282	Micro-retrognathia	NR	Present: Thoracic scoliosis and posterior rib gap defects	Gastrostomy, multiple respiratory decompensations, chronic respiratory failure	
	SNRPB (NM_198216, NM_003091)	g.2447953C>A c.164G>T p.Arg55Met	NS	#117650	#182282	Micro-retrognathia	NR	Present: Thoracic scoliosis and posterior rib gap defects	NR	
	SNRPB (NM_198216, NM_003091)	g.2447847G>T c.213+57C>A	NS	#117650	#182282	Micro-retrognathia	NR	Present: Posterior rib gap defects	Feeding difficulties requiring gastrostomy. Multiple orofacial interventions. Mixed hearing loss	
	SNRPB (NM_198216, NM_003091)	g.2447953C>G c.164G>C p.Arg55Thr	NS	#117650	#182282	Micro-retrognathia	NR	Present: Posterior rib gap defects	Multiple orofacial interventions, respiratory infections, chronic respiratory failure. Conductive hearing loss	

Basart et al., 2015	Unbalanced translocation	t(6;9)(p21.3;q22)	P	-	-	Micrognathia	Cleft	Unknown	NR		
	Deletion	Del 13q21.33q22.2 (6.1 Mb)									
	Deletion	Del 13q22.3 (800kb)									
	Deletion	Del 4p16.3 (~1.5Mb)	P	#194190	-	Micrognathia	Cleft	Present, however not specified	NR		Wolf-Hirsch-horn syndrome
	Balanced translocation	t(8;17)(q24.12;q24)	PP	-	-	Micrognathia	Cleft	Unknown	NR		
	Deletion	Del X (X0)	P	-	-	Micrognathia	Cleft	Unknown	NR		Turner syndrome
	Deletion	Del 9q24.1 (1.1 Mb)	LP	-	-	Micrognathia	Cleft	Unknown	NR		
	Unbalanced translocation	t(14;16)(p11;p12.3)	P	-	-	Micrognathia	Cleft	Unknown	NR		
	Deletion	Del 14p11-1pter (~200-400 Mb)									
	Duplication	Dup 16pter-16p12.3 (19.3 Mb)									
	Deletion	Del 5p15.33p14.3 (20.1 Mb)	P	#123450	-	Micrognathia	Cleft	Present, however not specified	NR		Cri-du-chat syndrome
	Deletion	Del Yq11.223q11.23 (2.4 Mb)	P	#192430	-	Micrognathia	Cleft	Present, however not specified	NR		22q11 deletion syndrome
	Deletion	Del 22q11.21q11.23 (2.9 Mb)									
	DAAM2	Del 6p21.2 (31 kb)	VUS	-	#606627	Micrognathia	Cleft	Unknown	NR		

	Duplication	Dup 1q21.1 (2.8 Mb)	VUS	-	-	Micrognathia	Cleft	Unknown	NR	
	Unbalanced translocation<br>Duplication<br>Deletion	t(13;21)(q12;q22)<br>Dup 13pter-13q12 (~20 Mb)<br>Del 21pter-21q22 (~25-45 Mb)	P	-	-	Micrognathia	Cleft	Unknown	NR	
	Unbalanced translocation<br>Duplication<br>Deletion	t(13;18)(q32.3;q21.33)<br>Dup 13q32.3-qter (~15 Mb)<br>Del 18q21.33-qter (~18 Mb)	P	-	-	Micrognathia	Cleft	Unknown	NR	
	Apparently balanced translocation	t(17;18)(p11.2;q21.1)	VUS	-	-	Micrognathia	Cleft	Unknown	NR	
	Deletion	Del 22q11.2 (1.5-3.0Mb)	P	#192430	-	Micrognathia	Cleft	Present, however not specified	NR	22q11 deletion syndrome
	SATB2	Del 2q33.1 (675 kb)<br>Dup 10q21.3 (730 kb)	P	-	#608148	Micrognathia	Cleft	Unknown	NR	
	Unbalanced translocation<br>Deletion<br>Duplication	t(1;15)(q42;q15)<br>Del 1q42 (~25 Mb)<br>Dup 15q15 (~62 Mb)	P	-	-	Micrognathia	Cleft	Unknown	NR	

	Unbalanced translocation<br><br>Deletion<br><br>Duplication	t(1;19)(p36.31;q13.42)<br><br>Del 1p36-pter (5.9 Mb)<br><br>Dup 19q13.4-qter (3.2 Mb)	P	-	-	Micrognathia	Cleft	Unknown	NR
	Unbalanced translocation<br><br>Deletion<br><br>Duplication	t(11;12)(q23.3;q24.3)<br><br>Del 11q23.3-qter (~15Mb)<br><br>Dup 12q24.3-qter (~12Mb)	LP	-	-	Micrognathia	Cleft	Unknown	NR
	Duplication	Dup 5q23.1 (2.1 Mb)<br><br>Dup Xp22 31 (1.6 Mb)	VUS	-	-	Micrognathia	Cleft	Unknown	NR
	Deletion	Del 4q31.3-q35 (~50Mb)	P	-	-	Micrognathia	Cleft	Unknown	NR
	Duplication	Dup 9p22.2-p22.3 (142b)	PP	-	-	Micrognathia	Cleft	Unknown	NR
	Duplication	Isodicentric duplication 15q13 (~10Mb)	P	-	-	Micrognathia	Cleft	Unknown	NR
	Duplication	Dup Xp21.1 (311 kb)<br><br>Dup 3p14.1 (234 kb)	VUS	-	-	Micrognathia	Cleft	Unknown	NR
	Deletion	Del 3q22.2-q22.3 (2.19 Mb)	LP	-	-	Micrognathia	Cleft	Unknown	NR

	COL2A1	NR	P	#108300	#120140	Micrognathia	Cleft	Present, however not specified	NR	Stickler syndrome type 1
	COL11A1	NR	P	#604841	#120280	Micrognathia	Cleft	Present, however not specified	NR	Stickler syndrome type 2
Basha et al., 2018	*GRHL3* (NM_198174.2)	c.1171C>T p.Arg391Cys	LP	#606713	#608317	NR	Submucous cleft	Unknown	NR	Van der Woude syndrome 2
Benko et al., 2009	*SOX9* (NM_000346)	75 kb deletion (chr17:66,175,000–66,250,000)	NS	-	#608160	Micrognathia	U-shaped cleft	Absent	Severe UAO requiring tracheostomy	
	SOX9 (NM_000346)	Centromeric deletion of >319 kb (chr17:65,730,750–66,049,600)	NS	-	#608160	Micrognathia	U-shaped cleft	Present: ASD	Normal oximetry	
	SOX9 (NM_000346)	Telomeric 36 kb deletion (chr17:69,153,000–69,189,000)	NS	-	#608160	Micrognathia	U-shaped cleft	Absent	Severe UAO requiring tracheostomy	
	SOX9 (NM_000346)	T>C mutation chr17:66,187,898	NS	-	#608160	Micrognathia	U-shaped cleft	Present: Mild septal hypertrophy and mild myopia	Severe UAO requiring tracheostomy and feeding difficulties requiring gastrostomy	
	SOX9 (NM_000346)	Translocation t(2;17)(q32;q24)	NS	-	#608160	Micrognathia	U-shaped cleft	Present: Mild cortical atrophy and transient hyperlaxity	Severe UAO requiring tracheostomy	
	SOX9 (NM_000346)	Translocation t(5;17)(q15;q24)	NS	-	#608160	Micrognathia	U-shaped cleft	Present: Astigmatism	Mild and transitory UAO	
	SOX9 (NM_000346)	Translocation t(2;17)(q24.1;q24.3)	NS	-	#608160	Micrognathia	U-shaped cleft	Present: Delayed bone age and retroesophageal artery	Severe UAO requiring tracheostomy and feeding difficulties requiring gastrostomy	

Bertola et al., 2018	*EIF4A3* (NM_014740.3)	Biallelic repeat expansion in 5'of UTR: 14repeats/16repeats	NS	#268305	#608546	Micro-retrognathia	Cleft	Present: Club feet, pectus deformity, supraglottic laryngeal anomalies, abnormal configuration of carpal and tarsal bones, absence of lower central incisors, low-set/posteriorly rotated ears, prominent ears, unfolded superior helix, mild hypoplastic thenar (regions), hypoplastic hallux, and triangular configuration of heels	Respiratory distress requiring tracheostomy and feeding difficulties requiring gastrostomy	Richieri-Costa-Pereira syndrome

	EIF4A3 (NM_014740.3)	Biallelic repeat expansion in 5'of UTR: 16repeats/16repeats	NS	#268305	#608546	Severe micro-retrognathia	Cleft	Present: Neonatal tooth, cleft mandible, narrow mouth, low-set and/or posteriorly rotated ears, unfolded superior helix, hypoplastic supraglottic structures/ epiglottis, mesomelia of upper and lower limbs, hypoplastic thenar, hypoplastic radius/ulna /tibia/fibula/hallux/bones of the 5th toes/1st metatarsal, shortening of 1st metacarpal bones, absence of 5th fingers, and preauricular pit	Respiratory distress requiring tracheostomy and feeding difficulties requiring gastrostomy. Developmental motor delay	Richieri-Costa-Pereira syndrome

	EIF4A3 (NM_014740.3)	Biallelic repeat expansion in 5'of UTR: 16repeats/16repeats	NS	#268305	#608546	Severe micrognathia	Cleft	Present: Cleft mandible, bilateral talipes equinovarus, absence of lower central and lateral incisors, low set and/or posteriorly positioned ears, prominent ears, pectus deformity, absent epiglottis, pulmonary branch stenosis, and hypoplastic hallux	Respiratory distress requiring tracheostomy and feeding difficulties requiring gastrostomy. Developmental motor delay and poor growth	Richieri-Costa-Pereira syndrome
Bhoj et al., 2013	Candidate genes: *BMP3*, *PRKG2*, *RASGEF1B*, *HNRNPD*, *HNRPDL*, *ENOPH1*, *SCD5*, *THAP9*	3.4 Mb deletion at 4q21.21q21.23 from 82,048,194 to 85,469,787	NS	-	-	Severe micrognathia	U-shaped cleft	Present: Small ASD, thin lip vermilion, long palpebral fissures, long philtrum, small hands and feet	Respiratory difficulties requiring MDO. Developmental delay and growth retardation.	4q deletion syndrome

Boschann et al., 2020	*TGDS* (NM_014305.3)	Homozygous missense variant, c.700T>C p.Tyr234His	P	#616145	#616146	Micro-retrognathia	Cleft	Present: Short stature, thin/arched eyebrows, proptosis, full cheeks, low-set ears, pectus deformity, joint hypermobility, clinodactyly of 5th finger, short long bones, genua valga, and hip dysplasia	Normal psychomotor development	Catel-Manzke syndrome
	TGDS (NM_014305.3)	Compound heterozygosity for the paternally inherited variant in exon 9, c.700T>C p.Tyr234His<br><br>and<br><br>Compound heterozygosity for the maternally inherited variant in exon 4, c.298G>A p.Ala100Ser	P	#616145	#616146	Micro-retrognathia	High palate, bifid uvula	Present: Short stature, thin/arched eyebrows, hypertelorism, low-set ears, short neck, joint hypermobility, clinodactyly of 5th finger, short long bones, and genua valga	Chronic lung problems during first months of life, due to paralysis of right-side diaphragm. Normal neurodevelopment.	Catel-Manzke syndrome

Braddock et al., 2016	Candidate gene: *RUNX1*	7.7 Mb deletion at chromosome 21q21.3q22.13 (covering bases chr21:31,391,467–39,118,687)	NS	#619980	#151385	Micrognathia	No cleft	Present: Large ears, small hands, peri-membranous VSD with aortic valve prolapse, lordosis, clubfoot, spastic diplegia, and thrombo-cytopenia	Growth deficient and no speech	Braddock-Carey syndrome
	Candidate gene: *RUNX1*	9.92 Mb deletion at chromosome 21q21.3q22.12 (covering bases chr21:26,968,514–36,909,426)	NS	#619980	#151385	Micrognathia	Posterior cleft	Present: Broad nasal root, thick everted vermilion of the lower lip, inverted U-shaped vermilion of the upper lip, lack of facial expression, large posteriorly rotated ears, enamel hypoplasia, microcephaly, anteriorly placed anus, clino- and camptodactyly, thrombocytopenia, ACC, and multicystic kidneys	Growth deficiency. Patient died at 5.5 years of age	Braddock-Carey syndrome

Camacho et al., 2020	*MYMK*	Missense variant, exon 3 c.271C>A p.Pro91Thr<br><br>and<br><br>Donor splice variant, intron 3 c.399 + 5G>A	P<br><br><br><br>NS	#254940	#615345	Micro-retrognathia	High arched palate	Present: Small nose, downslanting palpebral fissures, low-set ears, bilateral facial palsy, generalized joint laxity evident, and global muscular hypoplasia	Feeding problems requiring an NGT for two months. Delayed motor development in first years of life and growth delay.	Carey-Finemann-Ziter syndrome
Capkova et al., 201	Candidate genes: *BMP4*, *OTX2*	7.7 Mb microdeletion at 14q22q23	P	-	-	Micrognathia	No cleft	Present: Hypoplasia nasal bone, polydactyly, anophthalmia, horizontal palpebral fissure, and lack of gyrification of brain	Pregnancy terminated at 21 weeks gestation	
Castori et al., 2016	17q24.3 upstream of *SOX9*	~1 Mb heterozygous deletion in coding region of *KCNJ16*, *KCNJ2*, *HCNE-F2*, *9CE4Z*, *SOX9cre1* regulatory elements	NS	#114290	#608160	Micrognathia	Incomplete cleft	Present: Small ASD, almond-shaped eyes, small and short nose, flat face, and microstomia	MDO	Acampo-melic campomelic dysplasia

	17q24.3 upstream of *SOX9*	~1 Mb heterozygous deletion in coding region of *KCNJ16*, *KCNJ2*, *HCNE-F2*, *9CE4Z*, *SOX9cre1* regulatory elements	NS	#114290	#608160	Retrognathia	Cleft	Present: Triangular and long face, prominent nose, absent ear lobes, microstomia, hypoplastic uvula, sloping shoulders, tapering fingers with small nails, short great toes, severely hypoplastic scapulae, a small and elongated chest with 12 shortened ribs, underdeveloped transverse pedicles of the upper lumbar vertebrae, underdeveloped ilia with dysplastic pelvis, overtubulated metacarpal bones, relatively long ulnae at their distal ends, and flaring of the distal end of the radii	Left mixed deafness	Acampo-melic campomelic dysplasia

	17q24.3 upstream of *SOX9*	~1 Mb heterozygous deletion in coding region of *KCNJ16*, *KCNJ2*, *HCNE-F2*, *9CE4Z*, *SOX9cre1* regulatory elements	NS	#114290	#608160	Retrognathia	Cleft	Present: Triangular and long face, microstomia, attached/hypo-plastic ear lobes, prominent nose, sloping shoulders, small nails of hands, tapering fingers, bell-shaped chest with 11 pairs of ribs, hypoplastic scapulae, absent left transverse process of T12, mildly underdeveloped ilia and middle and distal phalanges of hands, overtubulated metacarpal bones, and a dysmorphic carpus with flaring of the distal end of ulnae and radii	Intellectual disability of mild degree and seizures	Acampo-melic campomelic dysplasia

	17q24.3 upstream of *SOX9*	~1 Mb heterozygous deletion in coding region of KCNJ16, KCNJ2, HCNE-F2, 9CE4Z, SOX9cre1 regulatory elements	NS	#114290	#608160	Retrognathia	Cleft	Present: Triangular and long face, microstomia, attached/hypo-plastic ear lobes, prominent nose, sloping shoulders, small nails of hands, slender fingers, short great toes, small and elongated chest with 11 pairs of ribs, underdeveloped transverse processes of T12 and upper lumbar vertebrae, small ilia, mild under-development of the middle and distal phalanges of hands, overtubulated metacarpal bones, and flaring of the distal end of the radii	Borderline cognitive impairment	Acampo-melic campomelic dysplasia

	17q24.3 upstream of *SOX9*	~1 Mb heterozygous deletion in coding region of KCNJ16, KCNJ2, HCNE-F2, 9CE4Z, SOX9cre1 regulatory elements	NS	#114290	#608160	Retrognathia	Cleft	Present: Borderline microcephaly, prominent nose, tapering fingers, camptodactyly of the 5th fingers, small nails of hands, short great toes, bilateral hallux valgus, fibular deviation of the 2nd and 3rd toes with overlapping of the 3rd toe over the 4th on the left	NR	Acampo-melic campomelic dysplasia
Daicheng et al., 2022	*RBM10* (NM_001204468)	Hemizygous frameshift mutation in exon 11, c.1113_1119del p.Ile372fs	NS	#311900	#300080	Micrognathia	Cleft	Present: Low-set ears, widely spaced eyes, sparse eyelashes, wide nasal bridge, wide mouth with downturned corners, single umbilical artery, permanent left SVC, and hypotonia	Severe respiratory distress and asphyxia requiring NICU admission, patient died 8 hours after birth	TARP syndrome

Davidson et al., 2012	Candidate genes: *MN1*, *NF2*	3.7 Mb deletion at 22q12.2 (base pairs 25937541 and 29635842)	NS	-	#156100 (*MN1*)<br><br>#607379 (*NF2*)	Micrognathia	Cleft	Present: Microcephaly, ocular hypertelorism, bilateral cerebello-pontine schwannomas, and multiple extramedullary/ intradural spine tumors	Multiple apneic episodes. Poor weight gain, necessitating NGT feeds. Mental retardation, delayed motor development and bilateral hearing loss	Neuro-fibromatosis type 2
De Lonlay-Debeney et al., 1998	Deletion	Del 11q21q23	NS	#224700	-	NR	NR	Present: Ebstein anomaly with small right ventricle, tricuspid insufficiency, and pulmonary stenosis. Coarse face, synophrys, hypertelorism, broad nasal bridge, low-set ears, right preauricular tag. Adducted thumbs, left renal malrotation, and bilateral agenesis of 12th ribs	Sucking and swallowing difficulties. Mild growth retardation and psychomotor retardation at age of 4 months	

	Trisomy	Trisomy of chromosome 11q resulting from maternal balanced translocation t(11;22)(q23;q11)	NS	#224700	-	NR	NR	Present: Ebstein anomaly with hypoplastic right ventricle and pulmonary atresia. Bilateral renal hypoplasia and adducted thumbs	Died of cardiac failure at 2 hours of life	
Demeer et al., 2018	*TBX22* (NM_016954)	c.356 + 1G>A	NS	-	#300307	Discrete micrognathia	Cleft	Unknown	NR	
Dupont et al., 2013	*COL2A1* (NM_001844)	Balanced translocation t(12; 15)(q13;q22.2)	NS	#108300	#120140	Micro-retrognathia	NR	Present: Round and flat face, proptosis, slight platyspondyly, and bilateral myopia	NR	Stickler syndrome type 1

Ehmke et al., 2014	*TGDS* (NM_014305.2)	c.892A>G p.Asn298Asp	NS	#616145	#616146	Severe micrognathia	V-shaped cleft	Present: Low-set ears, prominent antihelices, bilateral radial deviation, clinodactyly of the 2nd digit, mild ulnar deviation of the 3rd and 4th finger, Manzke dysostosis, short toes, short humeri, short femora, adducted thumbs, feet edema, short neck, wide fontanelle	Intubation and ventilation	Catel-Manzke syndrome
	TGDS (NM_014305.2)	c.270_271del p.Lys91Asnfs*22	NS	#616145	#616146					Catel-Manzke syndrome

TGDS (NM_014305.2)	c.298G>T p.Ala100Ser	NS	#616145	#616146	Micrognathia	U-shaped cleft	Present: Bilateral hand abnormalities, mild hypertelorism, upslanting palpebral fissures, thin eyebrows, VSD, triangular-shaped bone inserted between the 2nd metacarpal and its proximal phalanx bilaterally causing radial deviation of the index fingers, on the left hand an additional pin-shaped ossification center visible	Mandibular distraction procedures	Catel-Manzke syndrome
TGDS (NM_014305.2)	c.294T>G p.Phe98Leu	NS	#616145	#616146					Catel-Manzke syndrome

	TGDS (NM_014305.2)	c.298G>T p.Ala100Ser	NS	#616145	#616146	Micrognathia	U-shaped cleft	Present: Narrow nostrils, thin arched eyebrows, full cheeks, hypertelorism, isolated patent foramen ovale of no hemodynamic significance, shortening and striking radial deviation of the index finger bilaterally (which resembled a 2nd thumb), two extra ossification centers at the base of the radially deviated index fingers	Prone positioning to relieve UAO. Feeding problems requiring NGT feeding. Postnatal growth retardation	Catel-Manzke syndrome

	TGDS (NM_014305.2)	c.298G>T p.Ala100Ser	NS	#616145	#616146	Micrognathia	Cleft of soft palate	Present: Mild hypertelorism, long and narrow nose, obstruction of nasolacrimal duct, ankyloglossia, clinodactyly V, genua valga, both hands showed slight shortening and z-shaped deformation of the index finger resulting in radial deviation at the base of metacarpo-phalangeal joint and ulnar deviation at the level of PIP and DIP, rhomboid-shaped accessory bone inserted between the 2nd metacarpal bilaterally and its corresponding proximal phalanx causing a marked radial deviation of the index finger	No serious breathing problems	Catel-Manzke syndrome

TGDS (NM_014305.2)	c.298G>T p.Ala100Ser	NS	#616145	#616146	Micrognathia	U-shaped cleft	Present: Clinodactyly V, pectus deformity, additional pin-shaped ossicle on its ulnar side, and marked radial deviation of the right index finger caused by a bipartite accessory bone at the metacarpo-phalangeal joint	NR	Catel-Manzke syndrome
TGDS (NM_014305.2)	c.298G>T p.Ala100Ser	NS	#616145	#616146	Micrognathia	High arched, bifid uvula	Present: VSD, joint hypermobility, thin eyebrows, long columella, low-set ears, dysplastic helices, small mouth, clinodactyly V, and bilateral M. Perthes	Bronchial hyperreactivity in early childhood	Catel-Manzke syndrome
TGDS (NM_014305.2)	c.700T>C p.Tyr234His	NS	#616145	#616146					Catel-Manzke syndrome

	TGDS (NM_014305.2)	c.298G>T p.Ala100Ser	NS	#616145	#616146	Micrognathia	U-shaped cleft	Present: Manzke dysostosis, joint hypermobility, brachy-metacarpia, and scoliosis	Postnatal growth retardation and hearing loss	Catel-Manzke syndrome
	TGDS (NM_014305.2)	c.269A>G p.Glu90Gly	NS	#616145	#616146					Catel-Manzke syndrome
Fukami et al., 2012	>1.16 Mb upstream of *SOX9*	Paracentric inversion on chromosome 17 (breakpoints at 17q21.31 and 17q24.3)<br><br>Microdeletion at 17q24.3q23? spanning from -4.15 to -1.16 Mb relative to *SOX9*	NS	-	#608160	Micrognathia	Posterior U-shaped cleft	Present: Bilateral clubfeet, dolichocephaly with hypoplasia of the facial bone, and mild hypoplasia of left scapula. Campomelia and tibial skin dimples were not observed	Respiratory distress requiring tracheostomy at 8 months of age. Feeding difficulties requiring medical intervention.	
Gerard-Blanluet et al., 2007	Deletion<br><br>Trisomy	Del Xp11.2p?22.3<br><br>Tel14q++	NS<br><br>LP	-	-	Micro-retrognathia	Cleft	Absent: No additional anomalies found with fetopathological analysis	Termination of pregnancy at 25 weeks	
Gerth-Kahlert et al., 2011	*COL2A1* (NM_001844.4)	Heterozygous mutation in exon 38, c.2478_2479delGA p.Glu826AspfsX16	NS	#108300	#120140	Micro-retrognathia	U-shaped cleft	Present: Midface hypoplasia, anteverted nares and epicanthus, persistent foramen ovale, and left duplex kidney	Required oxygen after birth, no resuscitation. Hospitalized for the first 3.5 months of life on account of poor bottle feeding. Bilateral dense preretinal and vitreous hemorrhages	Stickler syndrome type 1

Gomez-Ospina and Bernstein, 2016	Deletion	Del 22q11 (3.3 Mb)	P	#192430	-	Micrognathia	Cleft	Present: Semicircular canal dysplasia	Unilateral sensorineural hearing loss	22q11 deletion syndrome
	Deletion	Del 18q22.3 (7.35 Mb)	P	#601808	-	Micrognathia	Cleft	Present: Congenital heart defect, toe syndactyly, bifid scrotum, and congenital aural atresia	Global development delay	
	Duplication	Dup 18p11.23 (7.9 Mb)		-	-					
	Deletion	Del 18q21.2 (25.11 Mb)	P	-	-	Micrognathia	Cleft	Present: Microcephaly, short stature, small corpus callosum, optic nerve hypoplasia, renal cyst, Peter's anomaly, and congenital cataracts	Global development delay, mixed hearing loss, epilepsy	
	Deletion	Del 16q11.2q12.2 (mosaic) (7.9 Mb)	LP	-	-	Micrognathia	Cleft	Present: VSD, ASD, and toe abnormalities	NGT feeding. Sensorineural hearing loss, growth retardation, and global development delay	
	Microdeletion	Microdeletion 16p11.2 (0.75 Mb)	LP	#611913	-	Micrognathia	Cleft	Present: Hypotonia	Autism and mixed hearing loss	16p11.2 deletion syndrome
	Microdeletion	Microdeletion 2p16.3 (0.188 Mb)	VUS	-	-	Micrognathia	Cleft	Absent	NR	
	Microdeletion	Microdeletion 2q13 (1.8 Mb)	VUS	-	-	Micrognathia	Cleft	Absent	Unilateral hearing loss	
	Microdeletion	Microdeletion 20q13.12 (0.657 Mb)								

	PGM1	Microdeletion 3p24.1 (0.057 Mb)	VUS	#614921	#171900	Micrognathia	Cleft	Present: Hypotonia, liver enzyme abnormalities	Global development delay	Congenital disorder of glyco-sylation
	SATB2	Microdeletion Xp22.31 (0.294 Mb)	VUS	#612313	#608148	Micrognathia	Cleft	Present: Strabismus, dysmorphic features, dental crowding, and hypotonia	Global development delay	Glass syndrome
	COL11A1	NR	NS	#604841	#120280	Micrognathia	Cleft	Present, however not specified	NR	Stickler syndrome
	TCOF1	NR	NS	#154500	#606847	Micrognathia	Cleft	Present, however not specified	NR	Treacher Collins syndrome
	MLL2	NR	NS	#147920	#602113	Micrognathia	Cleft	Present, however not specified	NR	Kabuki syndrome
	EFTUD2	NR	NS	#610536	#603892	Micrognathia	Cleft	Present, however not specified	NR	Mandibulo-facial dysostosis Guion-Almeida type

Gopakumar et al., 2014	*SOX9*	Heterozygous missense mutation within codon 176, p.P176L	NS	#114290	#608160	Severe micrognathia	Midline cleft	Present: Midface hypoplasia, flat nasal bridge, anteverted nares, short long bones in upper and lower extremity, hypoplasia of both scapula, 11 ribs, straight long bones, and advanced bone age	Mild respiratory distress	Acampo-melic campomelic dysplasia
Gordon et al., 2014	*SOX9*	280 kb deletion at 1,156 kb upstream of the SOX9 transcription start site	NS	-	#608160	Micrognathia	Cleft	Present: Crowded teeth, flat facial features, marked pectus deformity, and hypoplastic clavicles	Failure to thrive	
	SOX9	280 kb deletion at 1,156 kb upstream of the SOX9 transcription start site	NS	-	#608160	Micrognathia	Cleft	Present: Flat facial appearance and mild pectus deformity	Failure to thrive	
	SOX9	6 kb deletion at 407 kb upstream of SOX9	NS	-	#608160	Micrognathia	NR	Present: Dupuytren's contracture	Adult-onset hearing loss	

	SLC39A11	6.78 kb duplication within an intron of SLC39A11 (563 kb downstream of the SOX9 transcription start site)	NS	-	#616508	Micro- and/or retrognathia	Cleft	Absent	NR
Gordon et al., 2017	*MED13L* (NM_015335.4)	De novo deletion of 278,879 bp deleting exons 1-13 (breakpoint boundaries of chr12:116,443,043–116,721,923)	NS	-	#608771	Micrognathia	High palate, posterior cleft	Present: Short neck, short and broad palms, metatarsus varus, hirsutism of the back, short and upslanted palpebral fissures, right-sided convergent strabismus, long nose with a broad nasal bridge, wide open mouth, posteriorly rotated ears with a prominent antihelical stem and antitragus bilaterally, global hypotonia, and a MRI showing white matter anomalies	Feeding difficulties and severe developmental delay (no spoken words)

MED13L (NM_015335.4)	Heterozygous essential splice site mutation, c.6226-1G>C	NS	-	#608771	Micro-retrognathia	High palate, no cleft	Present: Stocky build, short neck, short and broad palms, and large feet with bilateral metatarsus varus. Short palpebral fissures, convergent strabismus, long nose with broad nasal bridge and tip, wide, open mouth with eversion of lower lip, posteriorly rotated ears with prominence of the antihelical stem. Micropenis, bilateral inguinal hernias. Generalized hypotonia. Hirsutism of the back. Brain MRI identified a thin corpus callosum, ventricular dilatation, and subcortical white matter anomalies	Feeding difficulties. Delay in motor and speech development

Gripp et al., 2011	*RBM10* (NM_005676.4)	Hemizygous, c.del159C p.Lys54SerfsX80	P	#311900	#300080	Micrognathia	U-shaped cleft	Present: Low-set and posteriorly angulated ears, brachycephalia, flat supraorbital ridges, upslanting palpebral fissure, mild telecanthus, cryptorchidism, cortical visual impairment, optic atrophy, mild myopia, chronic kidney disease, persistent left SVC, absence of right SVC, ASD, brachy-clinodactyly of 5th digit bilaterally, distal flexion crease of right 4th finger missing, toes mild syndactyly of 2nd + 3rd digits bilaterally.<br>MRI: heterotopia along lateral ventricles, cerebellar vermis hypoplasia and megacisterna magna	Respiratory distress requiring intubation and tracheostomy. Feeding problems requiring gastrostomy tube and Nissen fundoplication. Bilateral sensorineural hearing loss	TARP syndrome

Houdayer et al., 2001	Deletion	Deletion due to unbalanced translocation, t(2;21), del 2(q32.3q33.2)	NS	-	-	Micrognathia	Cleft of soft palate and posterior 1/3rd of hard palate	Present: Downslanted palpebral fissures, hypertelorism, short and hypoplastic midface, long philtrum, low-set ears, dysplastic pinnae, hyper-folded helices, absent superior branches of antihelices, narrow external auditory cannals, short neck, short first toes	Respiratory distress with oxygen desaturation

Higuchi et al., 2017	*COL2A1*	De novo heterozygous missense mutation, c.1142 G>A p.Gly381Asp	P	#108300	#120140	Micrognathia	Submucous cleft	Present: Bilateral leukocoria, ocular hypertelorism, shallow eye sockets, flat midface, depressed nasal bridge, anteverted nares, low-set ears, limitations in bilateral elbow flexion, radiograph showing thickening of calvaria, widening of distal humeral metaphysis, lack of femoral head ossification, deformity of femoral neck, distal femoral and proximal tibial epiphyseal ossification centers	Speech delay. Normal mental development	Stickler syndrome type 1

Højland et al., 2018	*RBM10* (NM_005676.4)	Hemizygous mutation in exon 4, c.273_283delinsA	LP	#311900	#300080	Micrognathia	High and narrow palate	Present: Hypotonia, ASD, thoracolumbar scoliosis, sloping forehead, prominent nasal bridge and nose, alveolar ridge overgrowth, high myopia, esotropia, displacement of lacrimal points, downslanted palpebral fissures, prominent supraorbital ridge, chin dimple, and incomplete cutaneous syndactyly of 2nd and 3rd toes	Feeding and breathing difficulties. Severe to profound intellectual disability. Unilateral hearing loss and absence of speech	TARP syndrome
Honey, 2016	*B3GALT6* (NM_080605)	Compound heterozygous mutations, c.[200C>T];[235A>G]	NS	#271640	#615291	Severe micrognathia	Cleft	Present: Contractures of his fingers, and multiple dislocations	Severe respiratory distress at birth, nursed in prone positioning. Died in the hospital at 1 month of age	Spondylo-epimeta-physeal dysplasia with joint laxity (SEMD-JL)

Hui et al., 2020	*ATN1*	Change in the histidine repeat region of ATN1	NS	#618494	#607642	Micrognathia	Cleft	Present: Hypotonia, arthrogryposis, craniosynostosis and cardiac abnormalities, small brainstem and cerebellum, with markedly prominent supratentorial and infratentorial subarachnoid spaces	UAO which required MDO and tracheostomy. Developed epilepsy at 1 month of life	CHEDDA syndrome
Indugula et al., 2022	*HIST1H1E*	De novo heterozygous variant, c.505_506insT p. Lys169IlefsTer27	NS	#617537	#142220	Mild micrognathia	Submucous cleft	Present: Frontal bossing, broad forehead, hypertelorism, bulbous nasal tip, full cheeks, high frontal hairline, macrocephaly, mild tracheomalacia, laryngomalacia, and mitral valve stenosis	Severe OSA requiring bilateral MDO (oAHI 31/h). Delayed motor development	Rahman syndrome

Izumi et al., 2012	Microdeletion	De novo 1.9 Mb deletion in 21q22.11	NS	#619980	-	Micrognathia	U-shaped cleft	Present: Hypotonia and dystonic movements, ASD, small muscular VSD, PDA, mild dilation of the right atrium and ventricle and elevated right ventricular systolic pressure, moderately enlarged pulmonary arteries, low anterior hairline, sparse eyebrows, downslanted palpebral fissures, small dimples on ear lobes	Respiratory distress requiring endotracheal intubation, chronic lung disease, and gastrostomy tube placement because of feeding problems	Braddock-Carey syndrome

Izumi et al., 2015	Duplication	19 Mb gain derived from chromosome 20 (10.6 Mb to 29.6 Mb), loss of heterozygosity in the chromosome 20 regions surrounding the copy number gain (mosaic SMC20 and maternal UPD20)	NS	-	-	Micrognathia	Cleft, high arched palate	Present: Relative macrocephaly, prominent forehead, frontal bossing, pectus carinatum, tall vertebral bodies, and long slender bones	Respiratory distress and feeding difficulties requiring gavage feeding. Developmental delay and growth retardation	
Jakobsen et al., 2007	*SOX9* *KCNJ2*	Balanced translocation t(2;17)(q23.3;q24.3) – 1,13 Mb upstream of SOX9, 800 kb downstream of KCNJ2	NS	-	#608160	Micrognathia	Cleft	Present: Flat face, broad nasal bridge, low-set ears and low-set hairline	Respiratory difficulties in early neonatal period. Pharyngeal flap surgery to decrease hypernasal speech	
	ZNF804B	Microdeletion 7q21.13 (0.4 Mb)	NS	-	-	Micrognathia	Cleft	Unknown	Respiratory difficulties in early neonatal period	
Jamshidi et al., 2004	Translocation	Balanced translocation t(2;17)(q24.1;24.3)	NS	-	-	Micrognathia	Cleft of soft palate	Absent	Moderate airway obstruction requiring prone positioning and NPA. Feeding difficulties requiring NGT feeds.	

Jezela-Stanek et al., 2009	Trisomy	Trisomy 22pter-q12.3	NS	#115470	-	Severe micrognathia	Cleft	Present: Unilateral renal agenesis, ectopia, numerous bilateral skin tags, bilateral coloboma of iris, ASD, persistent foramen ovale, downslanting palpebral fissures, hypertelorism, preauricular pit, microtia, and generalized hypotonia	Respiratory insufficiency. Delayed mental development	Cat-eye syndrome
Jiang et al., 2020	Candidate genes: *ORC1*, *SCP2*, *DAB1*	10 Mb microdeletion at 1p33p32.2	NS	-	#601902 (ORC1)<br><br>#184755 (SCP2)<br><br>#603448 (DAB1)	Micrognathia	High palate	Present: Short stature, leuko-encephalopathy, craniofacial dysplasia, and microcephaly	Developmental delay, speech delay, intellectual disability, and growth retardation	

Johnston et al., 2013	*RBM10* (NM_005676.4)	c.448C>T p.Gln150X	LP	#311900	#300080	Micrognathia	U-shaped cleft	Present: Broad and wide nasal bridge, short palpebral fissures, small ears with simple helices and prominent antihelices, mild pectus excavatum, unilateral single transverse palmar crease, cutaneous toe syndactyly and unilateral postaxial toe polydactyly, sacral dimple, horseshoe kidney with mild hydronephrosis, bilateral superior venae cavae, PDA, small secundum ASD, and CNS abnormalities (small frontal horns, immature cortex, abnormal corpus callosum, vermis, and cerebellar hemispheres)	Obstructive and central sleep apnea with hypoxemia. Died at 2,5 months of age secondary to respiratory failure	TARP syndrome *without talipes equinovarus*

Keeling et al., 2001	Deletion	De novo interstitial deletion, del 4q33.4q34.4	PP	-	-	Micrognathia	Complete cleft of secondary palate	Present: Hypoplastic left arm with shortened humerus, absent ulna, pterygium on left elbow preventing extension of elbow, absent 3rd, 4th, 5th digits of left hand, and clinodactyly of 5th digit on right hand. Anteverted nares, smooth philtrum, mild left facial hemi-hypertrophy, mild plagiocephaly and epicanthic folds. Patent foramen ovale.	Delayed overall development	4q deletion syndrome
Kekis et al., 2016	Trisomy	Mosaicism for trisomy 3 (47,XX,+3)	NS	-	-	Micrognathia	Cleft	Present: Dysplastic ears, prominent eyes, downturned corners of the mouth, deviated septum, high and convex nasal bridge, hypoplastic alae nasi, bilateral flexible flat feet	Asthma, hypernasal voice, bruises easily, and joint pain without joint dislocation or fracture	Trisomy 3 mosaicism

Knapp et al., 2019	*DPF2* (NM_006268.4)	De novo missense variant, c.1066T>G p.Cys356Gly	NS	#135900	#601671	Micrognathia	Cleft	Present: Right sided diaphragmatic hernia, frontal bossing, malar hypoplasia, infraorbital creases, low-set ears, and 5th finger and toenails subtly hypoplastic	Mandibular advancement surgery	Coffin-Siris syndrome
Kohmoto et al., 2016	*COL11A1* (NM_001854.3)	Heterozygous missense mutation in exon 61, n.4838G>A c.4520G>A p.Gly1507Asp	PP	#604841	#120280	Micro-retrognathia	U-shaped cleft	Present: Craniofacial anomalies. However, not described	Bilateral mild high-frequency sensorineural hearing loss	Stickler syndrome
Kramer et al., 2000	Deletion	Del 2q33-q35	NS	-	-	Retrognathia	Cleft	Present: Microcephaly, low-set ears, high receding forehead, prominent nasal bridge, deep palmar fissures. VSD, dilated pulmonary arteries, and lung hypoplasia. Strabismus, glaucoma, corneal clouding, and myopia	Intubated and ventilated after birth, followed by oxygen supply. Feeding difficulties requiring NGT feeds. Psychomotor and growth retardation	

Kumps et al., 2021	*RBM10* (NM_001204468.1)	Frameshift variant, c.413_416del p.Ser138Trpfs*60	NS	-	#300080	Retrognathia	No cleft	Present: Reduced suck reflex, nystagmus, hypotonia, myoclonus, a VSD, mild telecanthus, upslanting of palpebral fissures, large mouth with eversion of lower lip, scarce hair, mild hirsutism on back and legs, visual impairment, and a MRI showing slightly asymmetric lateral ventricles, bifronto-temporal cortical thinning, broad sulci and insulae, and hypoplasia of optic nerves	Recurrent respiratory infections, apneas, tube feeding. Growth delay and severe cognitive delay

Kylat, 2018	Microduplication	Duplication, dup 22q11.21 (728 Kb), paternally inherited	NS	#608363	-	Severe micrognathia	Posterior cleft	Absent: No other abnormalities were detected and the patient had a normal echocardiogram	UAO requiring prone positioning followed by MDO. Feeding difficulties requiring gastrostomy tube	
Lane et al., 2023	*RBM10*	De novo splicing mutation, c.2295+1G>A	PP	#311900	#300080	Micrognathia	NR	Present: Central hypotonia, low-set ears, hypertelorism, large fontanelle, crossed fused ectopic left kidney, absent right kidney, and ASD	Respiratory distress requiring NICU admission	TARP syndrome
Lecointre et al., 2009	517 to 1.477kb upstream of *SOX9*	Heterozygous deletion, del 17q24 (960 Kb)	NS	#114290	#608160	Micro-retrognathia	Cleft	Present: Midface hypoplasia, long philtrum, small scapulae, 11 pair of ribs, defective ischio-pubic ossification without bowing of the long bones, small patella, sandal gap, epiphyseal dysplasia of femoral head, thoracic spinal anomaly, scoliosis, short stature,	Mild articulation disorder and mild VPI	Acampo-melic campomelic dysplasia

								hypermetropia, astigmatism, and bilateral gonadectomy due to dysgenetic gonads		
Loewenthal et al., 2015	*PGM1*	c.112A>T p.Asn38Tyr	NS	#614921	#171900	Micrognathia	Cleft	Present: Short stature	Tracheostomy because of UAO. Increased serum transaminases, hypocortisolemia, low serum pseudocholinesterase. Learning disability	PGM1 deficiency
Lumaka et al., 2012	*BMP4*	Microdeletion 14q22.1–22.2 (2.79 Mb)	NS	-	#112262	Retrognathia	Cleft	Present: Bilateral congenital microphthalmia, sclerocornea, post-axial polydactyly, joint hyperlaxity, and a MRI showing bilateral white matter lesions in the corona radiatae and centrum semi-ovale	Growth delay, and delayed motor and mental development	

Lynch et al., 2014	*SNRPB*	g.2447952 C>G	NS	#117650	#182282	Micrognathia	Cleft	Present: Bell-shaped thorax and multiple posterior rib gaps	Feeding problems requiring gastrostomy. Oxygen therapy at home in infancy. Learning disability
	SNRPB	g.2447952 C>G	NS	#117650	#182282	Micrognathia	High arched	Present: Bell-shaped thorax, multiple posterior rib gaps, 11 rib pairs, downslanting palpebral fissures, and hypoplastic L5 pedicles	Feeding problems requiring gastrostomy. Mild learning disability
	SNRPB	g.2447847 G>T	NS	#117650	#182282	Micrognathia	Cleft	Present: Bell-shaped thorax, multiple posterior rib gaps, and downslanting palpebral fissures	Restrictive lung disease. Conductive hearing loss
	SNRPB	g.2451408 C>T	NS	#117650	#182282	Micrognathia	Cleft	Present: 5 pairs of poorly ossified ribs 2 longer ribs on left side, poorly ossified scoliosis, ASD, cystic hygroma, choanal atresia, multiple ptrygia, and nuchal webbing	NR

	SNRPB	g.2447952 C>G	NS	#117650	#182282	Micrognathia	High, short	Present: Abnormal thorax	NR
	SNRPB	g.2447952 C>G	NS	#117650	#182282	Micrognathia	Cleft	Present: Multiple posterior rib gaps and scoliosis	Conductive hearing loss. Growth hormone treatment
	SNRPB	g.2447951 C>G	NS	#117650	#182282	Micrognathia	Cleft	Present: Thin left 4th rib, scoliosis, and anal stenosis	Mild cognitive delay
	SNRPB	g.2447951 C>G	NS	#117650	#182282	Micrognathia	Cleft	Present: Bell-shaped thorax, multiple posterior rib gaps, 10 rib pairs, ASD, and echogenic kidneys	Thoracic insufficiency, NGT feeding
	SNRPB	g.2447952 C>G	NS	#117650	#182282	Micrognathia	Cleft	Present: Multiple posterior rib gaps, 10 rib pairs, cleft lip, and scoliosis	Tracheostomy
	SNRPB	g.2447951 C>A	NS	#117650	#182282	Micrognathia	Cleft	Present: Bell-shaped thorax, multiple posterior rib gaps, ASD, strabismus, and hyperopia	Feeding difficulties requiring gastrostomy. Conductive hearing loss

	SNRPB	g.2447846 G>A	NS	#117650	#182282	Micrognathia	Cleft	Present: Bell-shaped thorax, multiple posterior rib gaps, thin ribs, and missing left 12th rib	Conductive hearing loss	
Manotas et al., 2021	*RBM10*	c.1877del p.his627Leufs*78	P	#311900	#300080	Micrognathia	High palate	Present: Dolichocephaly, wide anterior fontanelle, hypertelorism, epicanthic folds, anteverted nostrils, low-set ears, long philtrum, sacral fossa, talipes equinovarus, persistent left SVC, large PDA, ASD, moderate pulmonary hypertension, horseshoe kidney, and a MRI showing grade II bilateral germinal matrix hemorrhages and hypo-myelination of auditory and visual pathways	Feeding difficulties requiring NGT feeds. Acute respiratory infection with torpid evolution, requiring mechanical ventilation, with subsequent severe respiratory failure and death. Delayed psychomotor development	TARP syndrome

Martinet et al., 2006	Balanced translocation	Biparental inheritance of balanced reciprocal translocation, t(17;20)(q21.1;p11.21)mat, t(17;20)(q21.1;p11.21)pat	NS	-	-	Retrognathia	Posterior cleft	Present: Hypoplastic nares, hypoplasia of right ventricle with muscular hypertrophy, endocardial fibroelastosis, hypoplastic lungs, dysplastic left kidney, bilateral pelvicalyceal dilatation, central nervous system periventricular heterotopias, right sided club foot	Pregnancy was terminated, NB parents were first cousins	
Micale et al., 2020	*COL11A1* (NM_001854)	De novo variant in intron 41, c.3168+5G>C	VUS	#604841	#120280	Micrognathia	Cleft soft palate	Present: Hypoplasia of corpus callosum, broad forehead with frontal bossing, ocular proptosis with bilateral buphthalmus and megalocornea, blue sclerae, midface hypoplasia, hypoplastic nose with anteverted nostrils, reduced ischiatic notch,	Respiratory distress due to UAO requiring NICU admission and MDO. Conductive hearing loss	Stickler syndrome type 2

								precocious ossification of the proximal femoral epiphyses, broad metaphyses, short phalanges, and platyspondyly		
Miller et al., 2020	*TGDS*	Compound heterozygous for p.Ala100Ser variant and p.His103Arg variant	LP	#616145	#616146	Micro-retrognathia	Submucous cleft	Present: Full cheeks, upslanting palpebral fissures, columella that extends beneath nares, low-set ears, ASD, VSD, hyperopia, and no Manzke dysostosis	OSA requiring MDO and CPAP. Gastric tube placement for feeding difficulties and pyloromyotomy for pyloric stenosis during infancy	Catel-Manzke syndrome

Moog et al., 2001	*SOX9*	Heterozygous missense mutation, c.865C>T p.His165Tyr	NS	#114290	#608160	Micrognathia	Cleft	Present: Midface hypoplasia, round face, slightly depressed broad nasal bridge, low-set ears. Severe trachea-bronchomalacia and abnormal course of innominate artery with compression of trachea. Mild shortness of limbs and slight bowing of lower legs, severe club feet, short 1st rays, no nails on 1st toes, hypoplastic nails on other toes. Broad, puffy hands with normal length, but short middle fingers and hypoplastic nails. Wide internipple distance, excessive skin, and joint hypermobility	Severe respiratory distress requiring artificial ventilation and tracheostomy at age of 3 months. GERD requiring Nissen fundoplication and PEG at age of 7 months	Acampo-melic campomelic dysplasia

Morozumi et al., 2018	Presumed *SOX9*	t(2;17)(p15;q24.2)	NS	#114290	#608160	NR	Cleft	Present: Midface hypoplasia, tetralogy of Fallot, 11 pair of ribs, and bilateral bowed radiuses	Premature thelarche and precocious pubarche at 6 years of age (Tanner 3). Increased level of testosterone by gonadoblastoma originating from dysgenetic gonads with Y-chromosome-related DSD	Campomelic dysplasia
Mouillé et al., 2022	*SATB2*	c.1197dup p.(Lys400*)	P	#612313	#608148	NR	NR	Present: Hypodontia and fracture of clavicle and humerus	Intellectual disability and speech disorder/delay	SATB2-associated syndrome (Glass syndrome)
	SATB2	Del 2q33.1 (minimum size of 29.114 bp)	P	#612313	#608148	NR	NR	Present: Macrodontia and fracture of vertebra and thigh bone	Autism spectrum disorder, intellectual disability, and speech disorder/delay	SATB2-associated syndrome (Glass syndrome)
	SATB2	c.955 C>T p.(Gln319*)	P	#612313	#608148	NR	NR	Present: Macrodontia and fracture of forearm	Autism spectrum disorder, intellectual disability, and speech disorder/delay	SATB2-associated syndrome (Glass syndrome)
	SATB2	c.658 C>T p.(Gln220*)	P	#612313	#608148	NR	NR	Present: Macrodontia	Autism spectrum disorder, intellectual disability, and speech disorder/delay	SATB2-associated syndrome (Glass syndrome)
	SATB2	Del 2q31.3 (7.78 Mb)	P	#612313	#608148	NR	NR	Present: Dental abnormalities	Intellectual disability and speech disorder/delay	SATB2-associated syndrome (Glass syndrome)

	SATB2	c.715C>T p.Arg239*	P	#612313	#608148	NR	NR	Present: Dental abnormalities	Intellectual disability and speech disorder/delay	SATB2-associated syndrome (Glass syndrome)
Murtaza et al., 2021	*SOX9*	Homozygous mutation, c.448C>A p.Pro150Thr	PP	-	#608160	Micrognathia	No cleft	Absent	Feeding and respiration concerns	
Nelson et al., 2011	*SOX9*	Heterozygous mutation, c.1312-1318del7ins5 p.Ser438ProfsX139	NS	#114290	#608160	Micrognathia	Cleft	Present: Mild midface hypoplasia, frontal bossing, downslanting palpebral fissures, mildly dysmorphic auricles with narrow external auditory canals bilaterally, short and bowed tibiae and fibulae, mild bowing of the femurs, bilateral hip dislocation, multiple hypoplastic right ribs, dextroconvex scoliosis of lower thoracic spine, and tracheomalacia	Respiratory distress requiring intubation and tracheotomy	Campomelic dysplasia

Nunes et al., 1994	Deletion	Del 7q21-q22	NS	#183600	-	Micrognathia	Submucous cleft	Present: Ectrodactyly and a low set left ear	Velopharyngeal insufficiency. Conductive hearing loss. Delayed language and motor development	
Omorodion et al., 2023	*RBM10*	Hemizygous maternally inherited variant, c.2038C>T p.Arg680*	P	#311900	#300080	Severe micro-retrognathia	High arched	Present: Sloping forehead, hypertelorism, small and upslanting palpebral fissures, absent eyelashes, broad nasal root with upturned nasal tip and alae, low-set ears, downturned corners of the mouth, digits held in flexion with marked 2nd finger clinodactyly, deep sacral dimple, hypotonia, abnormal eyelid lacrimal punctum bilaterally, small optic nerves, preretinal hemorrhages, stage I retinopathy of	Intubation and NICU admission. Passed away at 38 days of age from respiratory failure following discontinuation of CPAP	TARP syndrome

								prematurity in left eye, patent foramen ovale, hypertrophied right ventricle, mildly dilated ascending aorta, and vitelline vascular remnant	
Ounap et al., 2005	Duplication	De novo, dup 2q13-q22	PP	-	-	Micrognathia	V-shaped cleft	Present: Bifid uvula, prominent ears, mild exophthalmos, epicanthal folds, convergent strabismus, upturned nose, broad nasal tip, long philtrum, thin upper lip, cryptorchid testes, hypertrophic cardiomyopathy, and bicuspid aortic valve	Intubation at birth. Feeding difficulties require tube feeds until 7 months, suspected celiac disease. Spastic tetraparesis and severe developmental delay

Owczarek-Lipska et al., 2022	*RBM10* (NM_005676)	Novel splice site variant c.17+1G>C p.?	NS	#311900	#300080	NR	Cleft	Present: Expanded renal pelvic calyx system on both sides with relatively little medulla-cortex differentiation, myoclonus, undescended left testicle, and a MRI showing a microcephalic brain, simplified gyral pattern, ventriculo-megaly, thickening of corpus callosum, pontine and vermis hypoplasia	Oxygen supplementation during sleep. Failure to thrive. GERD and gastric tube feeding. Developmental disorder	TARP syndrome

Parmeggiani et al., 2017	Deletion	Del 6q13q14.1 (9.6 Mb)<br><br>and<br><br>Del 6q21q22.31 (11.2 Mb)	NS	-	-	Micro-retrognathia	NR	Present: Trigonocephaly, hypoplastic superciliary arches, sparse eyebrows, upslanting palpebral fissures, bilateral epicanthus, bilateral strabismus, telecanthus, asymmetric simple and low-set ears, long and smooth philtrum, thin upper and lower lip, postaxial polydactyly of the left hand and both feet, small VSD, dysplastic aortic valve, and aortic insufficiency, micropenis with cryptorchidism, and generalized hypotonia	Respiratory distress and cyanosis at birth requiring oxygen therapy, subsequently tracheostomy was performed at 17th day of life followed by MDO at 4 months of age. Feeding difficulties requiring a feeding tube. Severe psychomotor development delay and intellectual disability

Pengelly et al., 2015	*IRF6* (NM_006147)	c.604G>A p.Val202Ile	NS	-	#607199	Micrognathia	Cleft	Present, however not specified	Ventilator support (ICU) at birth, mandibuloplasty	
Pferdehirt et al., 2015	*TGDS*	Homozygous variant c.298G>T p.Ala100Ser	P	#616145	#616146	Retrognathia	Narrow arched with small groove of the posterior soft palate	Present: Pharyngo-malacia, laryngomalacia, tubular-appearing nose with high nasal bridge and pinched nares, ankyloglossia, long fingers and toes, index fingers deviated and overlapping, mild pectus deformity, and an X-ray showing hypoplastic 2nd proximal phalanges, accessory bone located at base of 2nd digit, medial deviation of distal 2nd digits	Mixed sleep apnea and respiratory insufficiency requiring tracheostomy. Gastroesophageal reflux and gastrostomy	Catel-Manzke syndrome

Preiksaitiene et al., 2016	*SOX9*	De novo heterozygous missense mutation in exon 1, c.316A>G p.Lys106Glu	NS	#114290	#608160	Micrognathia	NR	Present: Hypotonia, flat face, low-set ears, shallow orbits, microstomia, short neck, small chest, congenital bilateral metatarsus varus deformities, congenital dislocation of the hips, clitoromegaly, trachea-bronchomalacia, scoliosis and kyphosis of the thoracic spine, 11 pairs of ribs, abnormal cervical vertebral bodies, and dysplasia of thoracic vertebrae	Respiratory distress, complicated respiratory infections, and feeding problems	Acampo-melic campomelic dysplasia
Prescott et al., 2016	*MED12* (NM_005120.2)	Hemizygous missense variant in exon 13, c.1862G>A p.Arg621Gln	NS	-	#300188	Micrognathia	Cleft	Present: Horizontal gaze paresis, anomalies of the inner ear, and a cervical block vertebra	UAO requiring tracheostomy. Mild to moderate intellectual disability	

Rainger et al., 2014	*SATB2*	De novo translocation, t(2;11)(q32;p14)	NS	-	#608148	Micrognathia	Cleft	Present: Long thin face with prominent nasal bridge, small mouth, bilateral arachnodactyly, oligodontia, bilateral strabismus, on MRI enlargement of left lateral ventricle, mild asymmetry of the cerebral hemispheres and cranium, and small anterior commissure	Feeding difficulties, speech delay, delayed psychomotor development, and significant intellectual disability
	SATB2	De novo translocation, t(2;3)(q33.1;q26.33)	NS	-	#608148	Micrognathia	Cleft	Present: Small mouth, prominent nasal bridge, long nose, long columella, arachnodactyly, and osteomalacia	Feeding difficulties, severe GERD and constipation. No speech, severe intellectual disability, and delayed psychomotor development

Ramieri et al., 2011	Candidate genes: *ATR, FOXL2*	Del 3q22.1-q25.2	NS	-	#601215 (*ATR*)<br><br>#605597 (*FOXL2*)	Retro-micrognathia	Cleft	Present: Bilateral inguinal hernia, ASD, arthrogryposis of the superior limbs and knees in association with clinodactyly, overlapping of 2nd and 3rd digits of both hands and feet, microcephaly, brachycephaly, round face, blepharon-phimosis, ptosis, epicantus inversus, microphthalmia, hypotelorism, broad nasal bridge, beaked nose, large and low-set ears, large mouth, and short neck with pterygium	Respiratory distress requiring NICU admission. Gastroesophageal reflux	Micro-deletion 3q syndrome

Richards et al., 2013	*COL11A1* (NM_001854.3)	c.1421dupC p.Gly475Argfs*9	NS	#604841	#120280	NR	NR	Present: Congenital and high myopia	Profound hearing loss	Stickler syndrome, type 2
	COL11A1 (NM_001854.3)	c.991-24A>G in intron 8	NS	#604841	#120280					
	COL11A1 (NM_001854.3)	c.1421dupC p.Gly475Argfs*9	NS	#604841	#120280	NR	NR	Present: however, not specified	Cochlear implants	Stickler syndrome, type 2
		c.991-24A>G in intron 8	NS	#604841	#120280					
Robbins et al., 2018	*IRF6*	Heterozygous novel variant, c.6682A>G	NS	#119300	#607199	Micrognathia	Cleft	Present: Tongue-palate fusion, interalveolar bands, and laryngomalacia	UAO requiring MDO	Van der Woude syndrome

Roberti et al., 2018	Microdeletion	De novo heterozygous microdeletion, del 12q13.2-q13.3 (500 Kb)	NS	-	-	Micrognathia	Cleft	Present: Peculiar face, triphalangeal thumbs, congenital peri-membranous ventricular defect, inherited hypo-regenerative anemia, prominent nose bridge, ear malformations, ocular asymmetry with buphthalmos, intermittent exotropia with left eye dominance, severe myopia, tooth decay and cavities, skeletal malformations; short stature, right-convex thoracic scoliosis with dorsal hump, hip dysmetria with heterometry of lower limbs, shortness and clinodactyly of fingers with hypoplasia of the distal	Neurodevelopmental delay, mental deficiency. Chronic middle ear otitis, bilateral mixed hearing loss. Rhinolalia and dysarthria	Diamond-Blackfan anemia, Klippel-Feil syndrome

								phalanges of 1st fingers, cutis laxa, endocrinological alterations, trigeminal nerve palsy, atlanto-occipital assimilation, arcuate foramen and occipito-condylar hyperplasia, foramen magnum was severely reduced, anomalies of external auditory canals, absent mastoid pneumatization, and abnormal course of the facial nerve bones canal	
Rossi et al., 2009	Deletion	De novo, del 4q34.1 (16.435 Mb)	NS	-	-	Micrognathia	NR	Present: Cardiac abnormalities, fluttering nystagmus, hypertelorism, clinodactyly of the left and right 5th toes, an in-toe gait, primary amenorrhea, and myopia	Respiratory compromise requiring tracheostomy. Learning disabilities

Sahoo et al., 2011	*BMP2*	Del 20p13p12 (592.7 kb)	NS	-	#112261	Micrognathia	Cleft	Present: Long philtrum, open posterior fontanelle, large anterior fontanelle, patent foramen ovale, deep palmar flexion creases, short 5th fingers with mild meso-brachydactyly bilaterally, clinodactyly, and pectus excavatum	NR
	BMP2	Del 20p13p12.2 (5.37 Mb)	NS	-	#112261	Micrognathia	U-shaped cleft (soft palate)	Present: Flat facial profile, downslanting palpebral fissures, low nasal bridge, small upturned nose with anteverted nares, pinpoint hemangioma nose, long philtrum, transverse crease across chin, delayed reflexes, significant central hypotonia, and lacrimal duct stenosis	Feeding difficulties
		Balanced translocation t(3;5)(q10;q10)	NS	-	#112261				

Saito et al., 2022	Candidate genes: *MN1, NF2*	De novo, microdeletion in chromosome 22q12	NS	#101000	#156100 (MN1)<br><br>#607379 (NF2)	Micrognathia	Bifid uvula	Present: Low-set ears, mild cleft lip, and multiple subcutaneous nodules, ~2–3 cm in diameter, with a smooth surface without discoloration were present on the left forearm, periumbilical region, and right thigh. MRI showing left-sided vestibular schwannoma and Chiari malformation type I, development of multiple spinal tumors and bilateral posterior semicircular canal hypoplasia (on MRI)	OSA. Motor, speech and language developmental delays, and intellectual disability. Mild bilateral hearing loss	Neuro-fibromatosis type 2

Salinero et al., 2020	Microduplication	Microduplication in 1q21.1q21.2 (896 kb)	NS	#612475	-	Micrognathia	Cleft of secondary palate	Present: Small ears, upturned nose, long philtrum, thin upper lip, pectus carinatum, small ASD, and hypotonia	Respiratory distress at birth (AHI 72/h, without central apnea), improving with prone positioning, requiring NICU admission and subsequently tongue-lip adhesion at 3 weeks of age. Feeding problems requiring gastrostomy tube placement. Recurrent ear infections requiring bilateral myringotomy with tympanostomy tube insertion. Developmental delay (speech)	1q21 micro-duplication syndrome
Sanchez-Castro et al., 2013	*SOX9* (NM_000346)	Del 17q24 (~1 Mb), –1.25 to –2.3 Mb upstream of SOX9	NS	-	#608160	NR	NR	Absent	NR	
	SOX9 (NM_000346)	Del 17q24 (~1 Mb), –1.25 to –2.3 Mb upstream of SOX9	NS	-	#608160	NR	NR	Present: Incomplete right bundle branch block, aortic valve insufficiency, mitral valve ballooning, mild tricuspid valve insufficiency, and pulmonary artery stenosis	NR	

	SOX9 (NM_000346)	Del 17q24 (~1 Mb), –1.25 to –2.3 Mb upstream of SOX9	NS	-	#608160	NR	NR	Present: Bicuspid aortic valve, hypertelorism, thin lips, posteriorly rotated and low-set left ear, and tubular nose	NR		
	SOX9 (NM_000346)	Del 17q24 (~1 Mb), –1.25 to –2.3 Mb upstream of SOX9	NS	-	#608160	NR	NR	Present: ASD and valvular pulmonary stenosis	NR		
Sangsin et al., 2016	*COL2A1* (NM_001844.4)	Heterozygous mutation, c.4161_4165del p.Gln1387His*fs30	NS	#183900	#120140	Micrognathia	Cleft	Present: Short stature, flattened face, short neck, umbilical hernia, platyspondyly, ossification of femoral head was absent, and long bones showed short broad tubular shape with metaphyseal flaring	NR		Spondylo-epiphyseal dysplasia congenita (SEDC)

Schoner et al., 2017	*TGDS* (NM_014305.3)	Two compound heterozygous mutations, c.298G>T p.Ala100Ser<br><br>and<br><br>c.895G>A p.Asp299Asn	NS	#616145	#616146	Retrognathia	U-shaped cleft	Present: Dolichocephaly, a broad forehead, widely spaced eyes, proptosis, a short nose with a depressed nasal bridge, a long philtrum, narrow mouth, full cheeks, short, low-set and posteriorly rotated ears, short frenulum, short neck with cutis laxa, narrow shoulder girdle, VSD, coarctation of the aorta, and anomalies of the middle fingers and halluces	Pregnancy was terminated at 21+2 weeks of gestation	Atypical Catel-Manzke syndrome

Sismani et al., 2015	Deletion	De novo, del 1q43q44	NS	#612337	-	Micro-retrognathia	Complete U-shaped cleft	Present: Microcephaly, tip of the tongue mildly bifid, clitoris appeared large, anogenital distance appeared shortened, ASD, neuroglial migration defects in the periventricular and subcortical cerebral white matter. Umbilical cord had 3 vessels, but one umbilical artery was seen to be collapsed, showing luminal occlusion and no evidence of blood flow at the fetal edge	Growth restriction. Pregnancy terminated at 28 weeks of gestation	1q43q44 deletion syndrome

Sleiman et al., 2017	*KIF15* (NM_020242.2)	Homozygous nonsense mutation, c.1501C>T p.R501*	NS	#619981	#617569	Micro-retrognathia	Cleft	Present: Microcephaly, microphthalmia, downslanting of palpebral fissure, bulbous nose, wide mouth, bilateral external auditory canal atresia, bilateral clinodactyly, and thrombo-cytopenia	Deafness and no spoken words	Braddock-Carey syndrome
Smyk et al., 2015	1.28 Mb upstream to *SOX9*	De novo ~1.58 Mb deletion in chromosome region 17q24.3 mapping ~1.28 Mb 5’ to *SOX9*	PP	-	#608160	Retrognathia	Cleft	Present: Small PDA and patent foramen ovale, diffuse osteopenia, several fractures, prominent forehead, sparse anterior hair, light blue sclerae, mildly depressed nasal bridge, full cheeks, narrow mouth with thin vermillion of the lips, faint nevus flammeus of the forehead, dental crowding, short 5th fingers, mild joint laxity, genu recurvatum, and pes planus	Respiratory distress (OSA) requiring 6 weeks NICU admission, mandibular osteotomies and distraction tracheostomy. Asthma. Gastroesophageal reflux and other feeding problems requiring gastrostomy tube placement and Nissen fundoplication	

Sood et al., 2021	Candidate gene: *TARP*	Del 7p14.1	NS	-	-	Micrognathia	Cleft	Present: Bilateral epicanthus folds and right temporal hyper-pigmented spot	NR
				-	-	Micrognathia	Cleft	Present: Ankyloglossia and bilateral clinodactyly	Difficulty in respiration
				-	-	Micrognathia	Cleft	Present: Bilateral low-set ears, VSD, thin and lax skin, bilateral index finger, and clinodactyly	NR
				-	-	Micrognathia	Cleft (posterior one third)	Present: Depressed right supraorbital margin, low-set ears, parrot beak nose, and left anterior mongoloid slant	NR
				-	-	Retrognathia	Cleft	Present: Bifid uvula and generalized enamel hypoplasia	NR
	Candidate genes: *NPS*, *FOX12*, *CLRN3*, *PTPRE*	Dup 10q26.2 (551 kb)	NS	-	-	Retrognathia	Cleft	Present: Bifid uvula, cleft fistula, and septal defect	NR
	Candidate gene: *DUSP22*	Dup 6p25.3	VUS	-	-	Retrognathia	Cleft	Present: Bifid uvula, ASD, and osteogenesis imperfecta	Respiratory difficulty

	Candidate genes: *TLR3*, *FAM149A*, *FLJ38576*, *CYP4V2*	Dup 4q35.1	NS	-	-	Micrognathia	Cleft	Present: Ankyloglossia and bilateral clinodactyly	Difficulty in respiration
	Candidate genes: *SLC2A14*, *SLC2A3*	Dup 12p13.31	NS	-	-				
	Candidate genes: *ADAM5*, *ADAM3A*	Del 8p11.22	NS	-	-	Micrognathia	Cleft	Present: Bilateral low-set ears, thin and lax skin, VSD, bilateral index finger, and clinodactyly	NR
	Candidate genes: *FAM74A7*, *SPATA31A6*, *CNTNAP3B*, *CNTNA3P2*	Dup 9p12-p11.2	VUS	-	-	Micrognathia	Cleft (posterior one third)	Present: Depressed right supraorbital margin, low-set ears, parrot beak nose, and left anterior mongoloid slant	NR
	Candidate genes: *SKI*, *MORN1*	Dup 1p36.33	VUS	-	-	Retrognathia	Cleft	Present: Bifid uvula and generalized enamel hypoplasia	NR
	Candidate gene: *SATB2*	Del 12q23.3	VUS	-	#608148				
	LOXL3 (NM_001289165)	c.653G>C p.Arg579Pro	P	-	#607163	Retrognathia	Cleft	Present: Bifid uvula, cleft fistula, and septal defect	NR
	SIX5 (NM_175875)	c.1271C>A p.Pro424His	VUS	-	#600963	Retrognathia	Cleft	Present: Bifid uvula, ASD, and osteogenesis imperfecta	Respiratory difficulty

Suemori et al., 2013	*COL2A1*	C>T that converted the codon for arginine-453(CGA) to a premature stop codon (TGA) at c.1750	P	#108300	#120140	Micrognathia	Cleft	Present: Saddle nose, myopia, bilateral membranous vitreous anomaly and circumferential perivascular retinal degeneration in right eye	NR	Stickler syndrome type 1
Sun et al., 2014	*CREBBP*	Unbalanced translocation, t(14;16)(q11.2; p13.13)	PP	-	#600140	Micrognathia	U-shaped cleft	Present: Bilateral congenital ptosis, upslanted and small eyes, bilateral inguinal hernias, umbilical hernia, bilateral clubfoot, and short fingers and toes	Respiratory difficulties requiring MDO. Developmental delay	
Sun et al., 2022	*ZC4H2* (NM_018684.4)	De novo, c.352C>T p.Gln118*	NS	#301041	#300897	Micrognathia	Cleft	Present: Severe arthrogryposis multiplex congenita, bilateral subependymal hemorrhage, and enlarged posterior horn of the left lateral ventricle	Feeding difficulties. Died two weeks after birth	Female-restricted Wieacker-Wolff syndrome

Takenouchi et al., 2014	*SOX9*	De novo missense mutation in exon 1, c.239T>G p.Val80Gly	NS	-	#608160	Micrognathia	Cleft	Present: Tracheomalacia, right talipes equinovarus, thin ribs, absent toenails, short ulnae, PDA, ASD (closed spontaneously), hypertelorism, downslanting palpebral fissures, hypoplasia of midfacial structures, severe myopia, narrow thorax, slender long bones and hip dislocation	Severe stridor with retraction and OSA, and multiple upper airway infections.<br>Severe developmental delay and severe deafness	
Takeshita et al., 2017	Deletion	Deletion in 1p36	NS	#607872	-	Micrognathia	Cleft	Present: Low anorectal anomaly, and hypoplastic auricle	UAO requiring NPA, intubation, ventilation, and subsequently tracheostomy, other interventions were ineffective.<br>Severe hearing loss	1p36 deletion syndrome
Tanpaiboon et al., 2010	*FOXC2*	De novo heterozygous frameshift mutation, c. 595-596insC	NS	#153400	#602402	Micrognathia	Cleft	Present: Bilateral mild ptosis, small mouth, crowding teeth, ankyloglossia, hydrocele, distichiasis, and lymphedema	NR	Lymph-edema-distichiasis syndrome

Taub et al., 2012	Deletion	Deletion, del(4)q33)(4qter-)	NS	-	-	Micrognathia	High arched palate with clefting of posterior portion	Present: Low-set unfolded ears, preductal coarctation, mild diffuse distal transverse aortic arch hypoplasia, mildly hypoplastic aortic and mitral valves, dilatation and hypertrophy of right ventricle, slight dilatation of pulmonary artery and mild tricuspid regurgitation	Respiratory distress requiring mandibular osteotomies	4q deletion syndrome
Tegay et al., 2009	*SATB2*	Balanced de novo translocation, t(2;14)(q33;q22)	NS	#217980	#608148	Severe micrognathia	NR	Present: Sparse eyebrows, puffy eyes, hypertelorism, full cheeks, small nose, low-set ears, short neck, 5th finger clinodactyly, laryngomalacia, absence of epiglottis, agenesis of corpus callosum with ventriculo-megaly, and osteopenia with fractures	Respiratory distress requiring intubation (OSA). Postnatal growth retardation and global developmental delay. Seizures	Toriello-Carey syndrome

Tooley et al., 2016	*SNRPB* (NM_198216)	g.2447952 C>T	NS	#117650	#182282	Micrognathia	Cleft	Present: Narrow thorax and multiple posterior rib gaps	Intermittent oxygen in the neonatal period. TSS requiring tracheostomy. NGT feeding. Conductive hearing loss
	SNRPB (NM_198216)	g.2447953 C>G	NS	#117650	#182282	Micrognathia	Cleft	Present: Redundant neck skin, widely spaced nipples, bell-shaped chest, scoliosis, multiple rib gaps, 7 ribs left side / 10 ribs right side, and right sided myopic astigmatism with anisometropia	UAO requiring nasopharyngeal CPAP followed by tracheostomy. Feeding difficulties and GERD requiring gastrostomy, and fundoplication. Mixed hearing loss. Growth failure. Died at age 10 from RSV-related pneumonia
Utami et al., 2014	*MED13L* (015335.4)	Balanced translocation, t(12;19) (q24;q12)	NS	#616789	#608771	Retrognathia	Cleft	Present: Multiple limb contractures, camptodactyly, metatarsus adductus of the thumb, bilateral equinovarus foot deformity, flat occiput, hypertelorism, flat philtrum, bulbous nose, broad nasal bridge, strabismus, hirsutism, and scoliosis	Moderate intellectual disability, global speech delay, absence seizures

Van-Landigham et al., 2008	Translocation	Unbalanced translocation, t(4;9)(q33;q33)pat	NS	-	-	Hypoplastic mandible	Posterior cleft	Present: Low-set ears, an accessory nipple, omphalocele, ulnar deviation of hands with clinodactyly, imperforate anus with fistula, sub-ependymal cysts and a slight prominence of the fourth ventricle, seizures, ASD, VSD, PDA, malrotation of large and small intestines, and unicornuate uterus lacking the left oviduct	Died at 4 days of age due to respiratory arrest

Velagaleti et al., 2005	Translocation ~900 kb upstream of *SOX9*	Balanced translocation, t(4;17)(q28.3;q24.3)	VUS	#114290	#608160	Micrognathia	Cleft	Present: Tracheostenosis, flat malar surfaces, depressed nasal bridge, prominent eyes, and 11 pairs of ribs. Left ear posteriorly rotated. Hypoplastic scapulae and iliac wings, irregularity of end plates of thoracic vertebrae, and anomaly of upper cervical spine	Tracheostomy, G-tube and fundoplication	Acampo-melic campomelic dysplasia
Walters-Sen et al., 2014	459-379 kb upstream of *SOX9*	t(6;17)(q25;q24)	NS	#114290	#608160	Hypoplastic mandible	Cleft	Present: Tracheomalacia, mild left hydronephrosis, cervical kyphosis and focal spinal stenosis at C4 level, hypoplastic scapulae, 11 rib pairs, infantile uterus, and lethargy	Central and obstructive apnea, NICU admission >6 months due to episodic oxygen desaturations with bradycardia, re-admission at 8 months after cardiopulmonary arrest secondary to aspiration event. NGT feeds and gastrostomy. Developmental delay	Acampo-melic campomelic dysplasia

Weaver et al., 2022	Mosaic trisomy	Mosaic trisomy 8, 47,XY,+8[26] /47,XYY[24]	P	-	-	Micrognathia	No cleft	Present, however not specified	UAO	
	Unbalanced translocation	Der(2)t(2;5) (q37.3;q35.3)	P	-	-	Micrognathia	No cleft	Present, however not specified	UAO	
	Deletion	Del 12q15q21.33	P	-	-	Micrognathia	No cleft	Present, however not specified	UAO	
	Deletion	Del 22q11 (chr22:17266915-19795050)	P	#192430	-	Micrognathia	No cleft	Present, however not specified	UAO	22q11 deletion syndrome
	Deletion	Del 22q11 (chr22:17255869-19800273)	P	#192430	-	Micrognathia	No cleft	Present, however not specified	UAO	22q11 deletion syndrome
	Deletion	Del 7q11.23 (chr7:72722981-74196360)	P	#194050	-	Micrognathia	No cleft	Present, however not specified	UAO	Williams syndrome
	Deletion	Del 22q11 (chr22:18886915-21463730)	P	#192430	-	Micrognathia	No cleft	Present, however not specified	UAO	22q11 deletion syndrome
	Mosaic trisomy	Mosaic trisomy (chr18:18539806-49926444)	P	#601161	-	Micrognathia	No cleft	Present, however not specified	UAO	Trisomy 18
	Unbalanced translocation	Der(3)t(3;5) (p26.2;q33.2)	P	-	-	Micrognathia	No cleft	Present, however not specified	UAO	
	Duplication	Dup 15q13.3 (chr15:32019919-32620127)	VUS	-	-	Micrognathia	No cleft	Present, however not specified	UAO	
	Deletion	Del 16p12.2 (chr16:21747738-22435811)	VUS			Micrognathia	No cleft	Present, however not specified	UAO	
	SATB2	Del 2q33.1 (chr2:200154449-200255711)	P	-	#608148	Micrognathia	Cleft	Present, however not specified	UAO	

	Deletion<br><br>Duplication	Del 9q34.3 (chr9:140859530-141048284)<br><br>Dup 7q31.1-q36.3 (chr7:107948471-159119736)	P	-	-	Micrognathia	Cleft	Present, however not specified	UAO	
	Unbalanced translocation	Der(4)t(3;4) (q27;q33)mat	P	-	-	Micrognathia	Cleft	Present, however not specified	UAO	
	SATB2	Del 2q32.2-q34 (chr2:190,300,735-213,638,953)	P	-	#608148	Micrognathia	Cleft	Present, however not specified	UAO	
	Deletion	Del 1q43-q44 (chr1:241625613-249202817)	P	-	-	Micrognathia	Cleft	Present, however not specified	UAO	
	Unbalanced translocation	Der(18)t(X;18) (p22.11; q22.3)	P	-	-	Micrognathia	Cleft	Present, however not specified	UAO	
	Deletion	Del 5q21.1-q23.2 (chr5:101570491-126436942)	P	-	-	Micrognathia	Cleft	Present, however not specified	UAO	
	Trisomy	Trisomy 12p (chr12: 191619-31733044)	P	-	-	Micrognathia	Cleft	Present, however not specified	UAO	
	Deletion	Del 4q32.3-q35.2 (chr4:166223992-190915650)	P	-	-	Micrognathia	Cleft	Present, however not specified	UAO	
	Duplication	Array N/A, trisomy 21	P	#190685	-	Micrognathia	Cleft	Present, however not specified	UAO	Trisomy 21
	Duplication	Dup 4q32.3-q33 (chr4:165035159-170380684)	P	-	-	Micrognathia	Cleft	Present, however not specified	UAO	
	Deletion	Del 7q21.2q22.3	P	-	-	Micrognathia	Cleft	Present, however not specified	UAO	

	Deletion	Del 8q24.23 (chr8:137704290-137850282)	VUS	-	-	Micrognathia	Cleft	Present, however not specified	UAO
	Duplication	Dup 1p13.3 (878 kb)	VUS	-	-	Micrognathia	Cleft	Present, however not specified	UAO
	Deletion	Del 3q26.1 (chr3:162526563-163076433)	VUS	-	-	Micrognathia	Cleft	Present, however not specified	UAO
	Duplication	Dup 16p13.13 (chr16:11450395-11702396)	VUS	-	-	Micrognathia	Cleft	Present, however not specified	UAO
	Deletion	Del 3q25.1	VUS	-	-	Micrognathia	Cleft	Present, however not specified	UAO
	Duplication	Dup 6q15-q16.1 (chr6:89927312-95680371)	VUS	-	-	Micrognathia	Cleft	Present, however not specified	UAO
Xu et al., 2016	*SOX9* (NM_000346)	Single heterozygous nucleotide substitution T>C in CNE1 in GATA-1 binding site	NS	-	#608160	Micrognathia	NR	Present: Talipes valgus and mild pectus excavatum	UAO
	SOX9 (NM_000346)	Heterozygous nucleotide substitution G>A in CNE3	NS	-	#608160	Micrognathia	NR	Present: Pectus excavatum and cholesteatoma left ear	UAO
	SOX9 (NM_000346)	Heterozygous nucleotide substitution C>T in CNE4	NS	-	#608160	Micrognathia	NR	Absent	UAO
	SOX9 (NM_000346)	Heterozygous nucleotide substitution G>C in CNE4	NS	-	#608160	Micrognathia	NR	Present: Bilateral dysplasia of hip, foot deformity, VSD, PDA, and vesicoureteral reflux	UAO

Xu et al., 2023	*LMNA*	De novo mutation, c.1968 + 3_1968+6delGAGT	NS	#176670	#150330	Micrognathia	High arched	Present: Sclerotic skin, dry skin, lipoatrophy, joint stiffness, prominent scalp veins, small ear lobes, and hair loss	UAO requiring bilateral MDO. Growth failure	Hutchinson-Gilford progeria syndrome
Yang et al., 2017	*BMPR1B* *GRM4*	Reciprocal translocation t(4;6)(q22;p21)	NS	-	#603248 / #604100	Micrognathia	Cleft	Present: Pectus excavatum and radioulnar synostosis	NR	
				-	#603248 / #604100	Micrognathia	High arched	Present: Pectus excavatum	NR	
				-	#603248 / #604100	Micrognathia	Cleft	Present: Pectus excavatum	NR	
	BMPR1B	Splicing mutation IVS2+2T>G	NS	-	#603248	Micrognathia	No cleft	Absent	NR	
				-	#603248	Micrognathia	Cleft	Present: Pectus excavatum	NR	
Yap et al., 2023	*ATP2B1* (NM_001366521.1)	Maternally inherited splice-site variant, c.3060+2 T>G<br>and<br>Paternally inherited missense variant, c.2938 G>T p.Val980Leu	NS	-	#108731	Micro-retrognathia	Cleft	Present: Unilateral talipes equinovarus, distinctive craniofacial appearance, periventricular heterotopia, brachy-mesophalangy, and cutaneous syndactyly	Persistent hypocalcemia from primary hypoparathyroidism. Neurodevelopmental and growth deficit	

Yekula et al., 2020	62 OMIM genes	Terminal deletion of chromosome 10q, band 10q26.11 to 10q26.3 (4.34 Mb)	NS	-	-	Micrognathia	Cleft	Present: PDA, mild dilation of aortic sinuses, clinodactyly of 5th finger and syndactyly involving the bilateral 2nd and 3rd toes, strabismus, sacral dimple, syrinx of conus medullaris (on MRI), and diffuse hypotonia	Airway obstruction. Global developmental delay	
Zechi-Ceide et al., 2013	*SLC26A2*	Compound heterozygous mutation, c.-26 + 2T>C<br><br>and<br><br>Missense mutation, c.862C>T p.Arg279Trp	NS	-	#606718	Mild micrognathia	U-shaped cleft	Present: Mild upper and lower limb shortness, short palpebral fissures, broad nasal root, posteriorly rotated ears, broad left helix, mildly proximally placed thumbs, brachydactyly, accelerated carpal ossification, metaepiphyseal dysplasia, genu valgum, and feet anomalies	Respiratory and feeding difficulties	Typical signs of DTD and rMED

	SLC26A2 (NM_000112)	Compound heterozygous mutation, c.-26 + 2T>C<br><br>and<br><br>Missense mutation, c.862C>T p.Arg279Trp	NS	-	#606718	Mild micrognathia	U-shaped cleft	Present: Mild upper and lower limb shortness, brachydactyly, accelerated carpal ossification, metaepiphyseal dysplasia, genu valgum, feet anomalies, and mild cystic swelling on the left external ear	Mild feeding difficulties	Typical signs of DTD and rMED

† As noted in the included studies: P = Pathogenic; LP = Likely Pathogenic; PP = Possibly Pathogenic; VUS = Variant of Uncertain Significance; NS = Not Specified.

‡ Other anomalies/clinically relevant findings as noted in the included studies: present – specified; present – not specified; absent; unknown.

Abbreviations: ACC = Agenesis of Corpus Callosum; ASD = Atrial Septal Defect; DSD = Disorders of Sex Development; DTD = Diastrophic Dysplasia; GERD = Gastro-Esophageal Reflux Disease; MDO = Mandibular Distraction Osteogenesis; NGT = Nasogastric Tube; NICU = Neonatal Intensive Care Unit; NPA Nasopharyngeal Airway; NR = Not Reported; OSA = Obstructive Sleep Apnea; OSMED = Otospondylomegaepiphyseal Dysplasia; PDA = Patent Ductus Arteriosus; rMED = Recessive form of Multiple Epiphysial Dysplasia; SVC = Superior Vena Cava; TSS = Toxic Shock Syndrome; UAO = Upper Airway Obstruction; VPI = Velopharyngeal Insufficiency; VSD = Ventricular Septal Defect.
